# Higher sequence diversity in the vaginal tract than in blood at early HIV-1 infection

**DOI:** 10.1371/journal.ppat.1006754

**Published:** 2018-01-18

**Authors:** Katja Klein, Gabrielle Nickel, Immaculate Nankya, Fred Kyeyune, Korey Demers, Emmanuel Ndashimye, Cynthia Kwok, Pai-Lien Chen, Sandra Rwambuya, Art Poon, Marshall Munjoma, Tsungai Chipato, Josaphat Byamugisha, Peter Mugyenyi, Robert A. Salata, Charles S. Morrison, Eric J. Arts

**Affiliations:** 1 Department of Microbiology and Immunology, University of Western Ontario, London, Ontario, Canada; 2 Department of Medicine, Case Western Reserve University, Cleveland, Ohio, United States of America; 3 Joint Clinical Research Centre, Kampala, Uganda; 4 FHI 360, Durham, North Carolina, United States of America; 5 Department of Pathology and Laboratory Medicine, University of Western Ontario, London, Ontario, Canada; 6 Department of Obstetrics and Gynaecology, University of Zimbabwe, Harare, Zimbabwe; 7 Faculty of Medicine, Makerere University, Kampala, Uganda; Emory University, UNITED STATES

## Abstract

In the majority of cases, human immunodeficiency virus type 1 (HIV-1) infection is transmitted through sexual intercourse. A single founder virus in the blood of the newly infected donor emerges from a genetic bottleneck, while in rarer instances multiple viruses are responsible for systemic infection. We sought to characterize the sequence diversity at early infection, between two distinct anatomical sites; the female reproductive tract vs. systemic compartment. We recruited 72 women from Uganda and Zimbabwe within seven months of HIV-1 infection. Using next generation deep sequencing, we analyzed the total genetic diversity within the C2-V3-C3 envelope region of HIV-1 isolated from the female genital tract at early infection and compared this to the diversity of HIV-1 in plasma. We then compared intra-patient viral diversity in matched cervical and blood samples with three or seven months post infection. Genetic analysis of the C2-V3-C3 region of HIV-1 *env* revealed that early HIV-1 isolates within blood displayed a more homogeneous genotype (mean 1.67 clones, range 1–5 clones) than clones in the female genital tract (mean 5.7 clones, range 3–10 clones) (p<0.0001). The higher *env* diversity observed within the genital tract compared to plasma was independent of HIV-1 subtype (A, C and D). Our analysis of early mucosal infections in women revealed high HIV-1 diversity in the vaginal tract but few transmitted clones in the blood. These novel *in vivo finding suggest a possible* mucosal sieve effect, leading to the establishment of a homogenous systemic infection.

## Author summary

During chronic HIV-1 infection, high viral diversity can be found in the blood and semen of donors. However, a single HIV-1 clone establishes productive infection in the recipient following heterosexual transmission. To investigate the genetic bottleneck occurring at the earliest stages of heterosexual HIV-1 transmission, we characterized the HIV-1 envelope sequence diversity at very early and early stages of infection in the female reproductive tract and matched plasma samples from a cohort of Ugandan and Zimbabwean women. A more diverse viral population was observed in the endocervical swab samples compared to plasma. Endocervical samples harbored a larger number of viral clones, while in the majority of plasma samples only a single clone was present early in infection. Interestingly, these observations were independent of HIV-1 subtype, hormonal contraceptive use or the number of sex acts and partners. Furthermore, in the cases of higher HIV-1 diversity in the blood during early infection, faster CD4 T cell decline were observed during chronic disease suggesting faster disease progression. Our findings provide novel *in vivo* evidence for the existence of an intra-patient genetic bottleneck restricting the HIV-1 from the vaginal tract to the blood during early heterosexual HIV-1 transmission.

## Introduction

The sexual transmission of HIV-1 establishes initial infection with high viral loads which is eventually resolved but followed by a slow progression to acquired immunodeficiency syndrome (AIDS). Male-to-female infection during vaginal intercourse is responsible for the majority of new HIV-1 infections. Despite the presence of a genetically diverse swarm of HIV variants in the blood and semen of infected donors, a single HIV-1 genetic variant (termed the transmitter founder or TF virus) emerges from an extreme “genetic bottleneck” to establish a systemic infection within the newly infected individual [1–3].

With different modes of transmission, the HIV-1 genetic sieve from donor to recipient differs with the greatest bottleneck observed from donor seminal or vaginal fluid to the vaginal or penile receptacle in the recipient. Direct blood-to-blood transmission shows evidence of a reduced genetic bottleneck due, in part, to simple mixing of donor virus and/or virally-infected cells with susceptible CD4+/CCR5+ cells, which may be enhanced through antigen-presenting cells (e.g. dendritic cells). In contrast, male-to-female heterosexual transmission of HIV-1 requires the inoculating virus from the semen to penetrate the vaginal mucosa, the epithelial layer, and then infect and/or bind to susceptible Langerhans or dendritic cells in the lamina propria of the vaginal/cervical lining [4, 5]. Each of these steps is poorly understood, e.g. penetration and infection of the various vaginal layers and dissemination into blood and lymphoid tissue could involve genetic bottlenecks. The entire process of heterosexual transmission leading to primary, disseminated infection to seroconversion only occurs in <1% of possible exposures [6, 7]. Certain viral characteristics such as glycosylation [8], receptor tropism [1], increased infinity for α4β7 integrin and general fitness [9] might favor selection of specific transmitted founder variants from the donor inoculum.

Following penetration of the vaginal tract mucosa, infectious foci within the lamina propria are generated by virus replicating in local mucosal CD4^+^ T cells and infiltrating T cells responding to the ensuing inflammatory environment [10, 11]. Primary SHIV and SIV infection studies in macaques suggest that this virus inoculation into the vaginal tract could result in multiple infectious foci in mucosa within a few hours after exposure [10, 11]. Based on a minimum of 12hrs for HIV-1 turnover, these infectious foci, if occurring during human primary infection, are likely the result of separate infections of different HIV-1 clones from the diverse HIV population from the donor inoculum. Current models propose that virus progeny from these foci are released and disseminated into the systemic circulation and draining lymph nodes [10] to establish the acute infection syndrome and high viral titers in the blood and other bodily fluids. Despite clear evidence that male-to-female transmission results in single transmitted HIV-1 clones found in blood (on average), there has not been extensive sampling in newly infected women to determine if a diverse HIV-1 population or a single HIV-1 clone replicates in the vaginal tract.

To date, the vast majority of research into the genetic bottle neck and breakthrough TF has been obtained by enrolling discordant transmission pairs, providing all measures to prevent transmission to each partner, and then anticipating accidental transmission events leading to new primary infections. Even with the ability to efficiently detect acute infections, obtaining time-matched samples from the genital tract and blood has proved challenging in many of these studies. As such, no study has compared or measured the HIV-1 genetic diversity in either the donor semen or recipient vaginal tract (e.g. in the case of male-to-female heterosexual transmission) compared with homogeneous HIV-1 population in the blood during acute or early HIV-1 infection. Here, we describe the variation in HIV-1 sequence diversity measured in the vaginal mucosal and systemic/blood compartments, at matched time points, within Zimbabwean and Ugandan women during very early (first three months) and early (three to seven month) HIV-1 infection. Specifically, we assessed the *env* sequence diversity of HIV-1 in endocervical swabs and blood samples by Next Generation deep Sequencing (NGS) analysis. Our results provide clear evidence for the existence of a diverse HIV-1 population that is maintained in the recipient female genital tract following transmission as well as a genetic bottleneck of the HIV-1 population from female genital mucosa to the blood during early infection.

## Results

### HIV-1 envelope sequences in blood plasma are more homogeneous than in the female genital tract during early stages of infection

**Fig 1:**
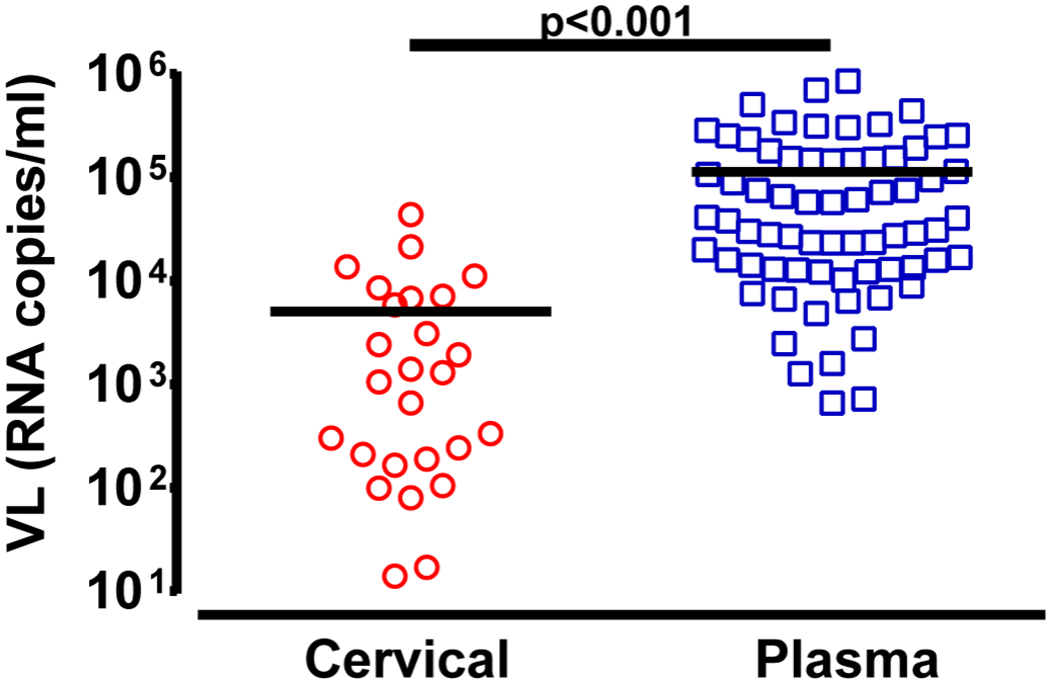
Viral loads in cervical and plasma samples early in infection. Viral RNA copies/ml were quantified in cervical (n = 29; red symbols) and plasma (n = 67; blue symbols) samples collected between 0–7 months of infection. Analysis of significance was done using a two-tailed Mann-Whitney test (p<0.001). Each symbol represents an individual subject.

**Fig 2:**
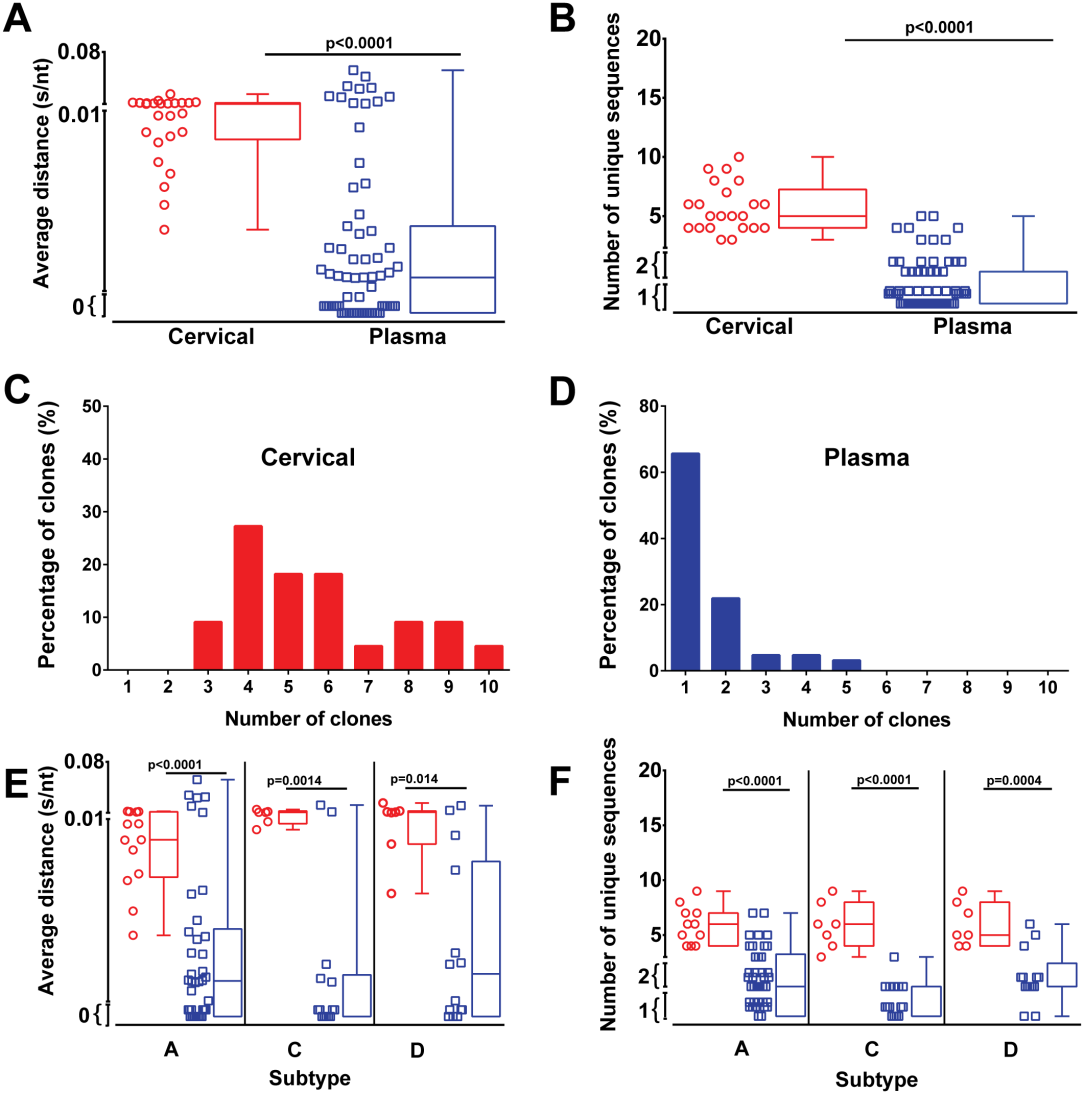
Higher HIV-1 diversity in the female genital tract than in blood within 7 months of infection. The HIV-1 C2-V3-C3 *env* region present in cervical (red symbols and red boxplot) and plasma (blue symbols and blue boxplot) samples were PCR amplified and analysed by NGS. The average p-distance (s/nt) of HIV sequences within C2-V3-C3 region were calculated using MEGA 6 with sequences below 200bp excluded from analysis (**A**). The number of unique sequences within each sample were identified using online Galaxy software to collapse all sequence reads for each sample (**B**). Frequency of clones in cervical (**C**) and plasma samples (**D**) were determined from the number of unique clones. Average distance (**E**) and the number of unique sequences (**F**) in cervical and plasma samples grouped by subtype of infection. Analysis of significance was done using a two-tailed Mann-Whitney test. Each symbol represents an individual subject.

Endocervical swab (n = 23) and blood (n = 67) samples were collected from 72 women within 7 months of HIV-1 infection. Viral load assessment revealed a significantly (p<0.001) lower mean viral load of 5,057 RNA copies/ml in cervical samples compared to mean viral load of 112,733 RNA copies/ml in plasma samples at the time point of sample collection (0–7 months) (Fig 1). To analyze the sequence diversity of the virus within both anatomical sites, the C2-V3 *env* region was PCR amplified followed by next generation sequencing. Neighbor joining and maximum likelihood trees were constructed and the average genetic p-distance was evaluated. Viruses from cervical swab samples showed a significantly (p<0.001) higher average genetic diversity of 0.010 substitutions/nucleotides (s/nt) (range 0.003–0.023 s/nt) while isolates from blood were genetically more homogenous with 0.005 s/nt (range 0.00–0.056 s/nt) (Fig 2A), representing a 2.2 fold lower diversity index within the infecting HIV-1 between the blood and endocervix. HIV genetic diversity was calculated based on all sequence reads in the patient sample population rather than just unique reads. This is best illustrated in nucleotide sequence alignment/highlighter plots (S1A–S1I Fig) where a single HIV-1 clone within a patient sample is shown as a black line in contrast to the grey lines showing multiple clones with specific mutations. The “thickness” of the lines in the highlighter plots provide the relative proportion of each clone within a patient sample/compartment. This feature of sequence reads is now being added into the highlighter plot algorithm provided by Los Alamos HIV Sequence Database and will provide a better tool for visualization of NGS data.

The general lack of genetic diversity of HIV in the blood during very early/early infection is consistent with previous reports [1, 2, 12–14]. We next analyzed the number of unique HIV-1 sequence reads obtained by NGS from the RT-PCR amplified HIV-1 RNA in cervical samples and blood plasma at very early/early infection. These analyses suggest a high number of HIV-1 clones in the cervical swab samples (6.68 sequences; range 3–10) compared to the numbers observed in plasma (1.67 sequences; range 1–5) (p<0.0001) (Fig 2B). It is important to note that an average of 1,264 cDNA copies (based on mean 5,057 copies/ml) of the endocervical swab were analyzed in the NGS run compared to a mean of 28,183 cDNA copies from the plasma (based on mean 112,733 RNA copies/ml).

Of particular note, we recorded only a single viral clone (0.0 s/nt, 1 unique sequence) in 32 out of the 67 plasma samples tested (47.7%) (Fig 2D). In contrast, infection of the endocervical samples involved an average of 6.03 unique clones and none of the very early/early endocervical samples had less than three unique HIV-1 clones (Fig 2C). For comparison, we also produced highlighter plots for the HIV quasispecies derived from plasma of eight chronically infected, untreated patients from Spain (all subtype B infected) (S1H and S1I Fig), sequences that were previously published [15]. In this study, we compared the results of deep sequencing the HIV V3 env region from chronically infected patient samples using four different NGS platforms: Illumina MiSeq, IonTorrent PGM, Roche 454, and PacBio RS. All technologies provided a very similar if not identical diversity pattern for reads >1% of the total (two standard deviations above the error rate). That being said, the sequencer-based error rates with the PGM, 454, and RS platforms was at least 10-fold greater than with the MiSeq platform but this did not impact reads >1% [15].

We next examined the type of nt substitutions in the quasispecies of these patient samples at early infection and again compared to the nt substitution types observed in plasma samples from chronic patients (S2A and S2B Fig). The frequency of the different types of HIV mutations was similar in the early plasma and cervical samples as well as in the chronic plasma samples. As expected, transition mutations were more frequent than transversion mutations. G-to-A mutations were most frequent in all disease states/samples which was likely impacted by both reverse transcriptase errors and cytidine deamination by ABOPEC3G. Approximately, 40 to 45% of G-to-A mutations in the HIV quasispecies of all disease states/samples were in the form of GG to GA which may have been mediated by ABOPEC3G [16–18]. When comparing ratio of nonsynonymous versus synonymous substitutions, the dN/dS was obviously lower for HIV analyzed in the early plasma versus early cervical or chronic plasma samples. However, when only comparing samples with diversity, the dN/dS ratios were remarkably similar with no statistical difference suggesting only a “weak” selection pressure.

### Absence of Y chromosome in cervical swabs

To exclude the possibility of HIV sequences being derived from repeated male donor exposure in these women, we performed DNA PCR for the X and Y chromosome on cervical swab samples. X chromosome was readily detected by PCR but only in 40% of the samples. Y chromosome could not be amplified by PCR in any of the 80 samples tested suggesting that donor semen was absent or only present at undetectable levels. Our swabs of the endocervix likely absorbed varying fractions of the submucosal layer. Ability to absorb the submucosal layer (with more cells) is dependent on mucosal thickness, which varies during the female cycle, i.e. reduced viscosity during ovulation to the thick cervical mucosal plug during the luteal phase. In addition, the border between the endocervix and ectocervix is not well defined and transitions from a thicker layer of cornified and non-cornified squamous epithelial cells to a thin single layer of columnar endothelial cells [19]. Given the variable levels of cellular X chromosomal DNA detected by PCR from the endocervical swab, we suspect a limited sampling of the submucosal layer, i.e. with the higher concentration of cells. Thus, the majority of HIV sampled in these swabs was likely shed into the mucosal layer. Previous studies have shown that Y chromosome from semen is readily detected in vaginal swabs by PCR and for up to 15 days post insemination [20–22]. Therefore, if semen and the inoculating HIV remained post coitus, we would have likely detected the Y chromosome, especially considering that semen composition is naturally formulated with putrescine, spermine, spermidine and cadaverine that intercalate and protect sperm DNA [23]. Semen nor sperm cells penetrate the submuscosal layer. Finally, the number of self-reported coital acts in a typical months prior to sample collection at very early/early infection did not correlate or remotely associate with HIV-1 sequence diversity or number of unique clones identified on the cervical swab (see below).

### The infecting HIV-1 subtype does not impact viral diversity at very early/early infection

Of the 23 cervical swab samples analyzed, 11 were of subtype A, 2 unknown/potential recombinants, and 6 each of subtype C and D, while 38 subtype A, 14 subtype C, 14 subtype D, 1 unknown/recombinant were identified in the very early/early plasma samples (S3 Fig). All Zimbabwean cohort samples were of subtype C while samples from the Ugandan cohort comprised of subtype A, C and D, as previously described [24]. Three samples (two from cervical and one from plasma) were identified as potential A/D recombinants but due to the short sequence, they remain unclassified or showed weak clustering with subtype B. This subtype distribution is in keeping with the expected geographical subtype distribution [25, 26]. The genetic diversity and number of unique *env* clones did not differ based on HIV-1 subtype, i.e. only one HIV-1 clone was observed in the majority of very early/early blood samples regardless of the infecting subtype A, C, or D (Fig 2E and 2F). Furthermore, the higher sequence diversity observed in cervical samples was not biased by a particular HIV-1 subtype.

### Comparing HIV sequence diversity to other possible contributing factors

Neither the use or the absence of hormonal contraceptive use, pregnancies, the number of sex partners, changing of sex partners, concurrent sex or the risk behavior of the partner or the participant herself were significantly associated (where sufficient sample numbers permitted statistical assessment) with higher genetic diversity or the number of unique HIV-1 sequences in the plasma or endocervix (S4A–S4G and S5A–S5G Figs). In relation to high concurrent sex with different partners (>2 partners/3 month), we had insufficient sample numbers for statistical analyses (S4D and S5D Figs). As described above, HIV-1 *env* genetic diversity and *env* clones in cervical or plasma samples were unassociated with the number of sex acts (0–214 acts) or the number of sex acts (0–210 acts) with condom use in the 3 months prior to sample collection during very early/early infection (S6A–S6H Fig).

**Fig 3:**
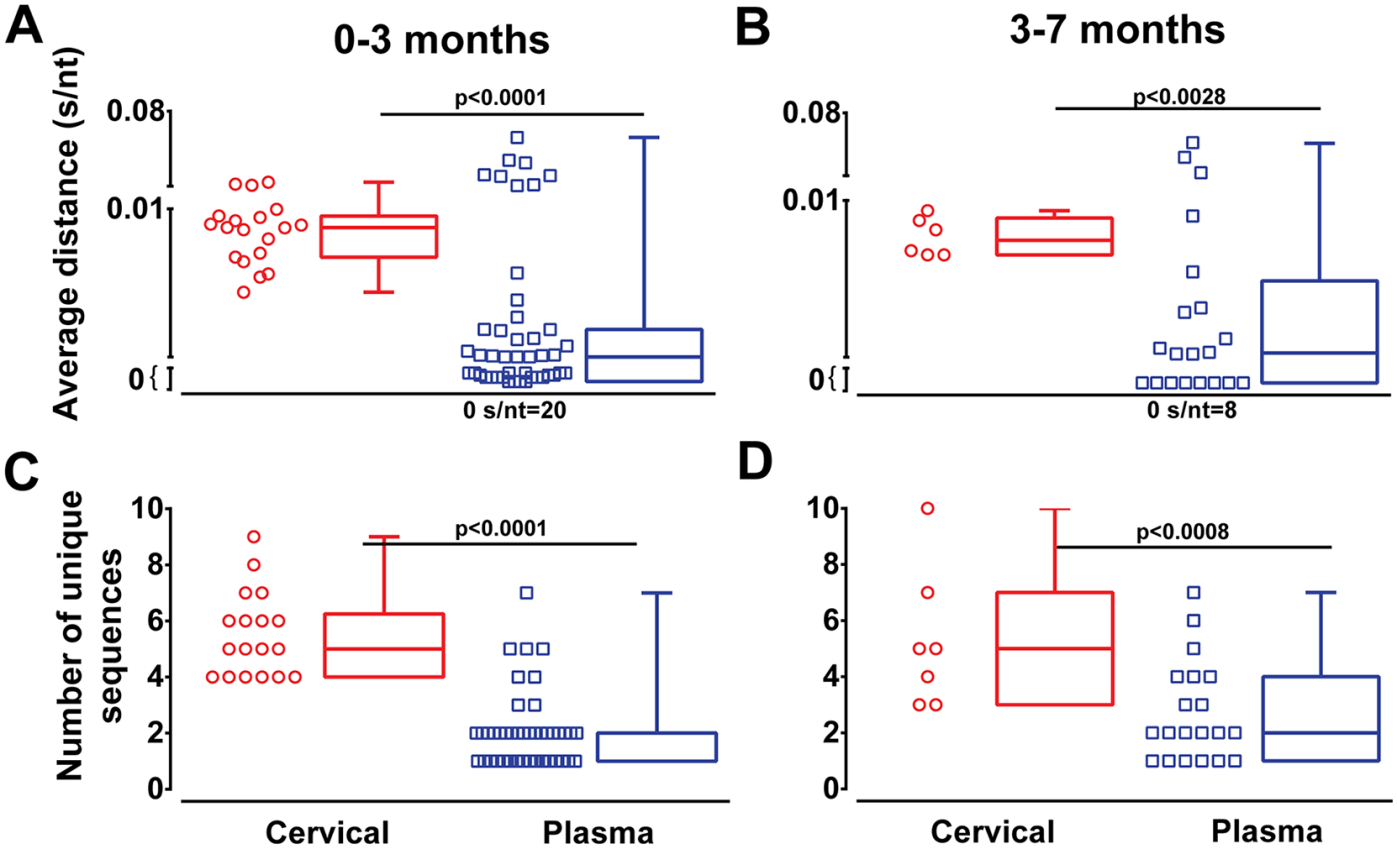
HIV-1 diversity in cervical samples and blood at very early and early infection. Average nucleotide p-distance (s/nt) and the number of unique sequences were analyzed following NGS. Average distance and number of unique sequences of cervical (red symbols and boxplot) and plasma (blue symbols and boxplot) samples were grouped into very early (0–3 months) and early infection (3–7 months) (**A-D**). Analysis of significance was done using a two-tailed Mann-Whitney test. Each symbol represents an individual subject.

Next we explored whether stratifying the patient samples into very early (0–3 months or ~Fiebig stage I-V) and early infection (3–7 months or ~Fiebig stage VI) could show stronger evidence for the observed genetic bottleneck. All sample groups were assessed by Grubbs Test (extreme studentized deviate, ESD) for the presence of significant outliers (Alpha ≥ 0.01) before analyses. This resulted in 19 cervical and 45 plasma samples available from the 0–3 period and 20 plasma and 6 cervical samples from the 3–7 months period. Higher *env* genetic diversity and larger numbers of unique sequences were evident (p <0.001) in cervical samples (0.009 s/nt and 5.5 sequences, respectively) compared to plasma (0.005 s/nt and 2.0 sequences, respectively) within the 0–3 months infection (Fig 3A and 3C). Within “early” 3–7 months infection, cervical HIV-1 genetic diversity (0.008 s/nt and 5.3 sequences respectively) was again higher than in the plasma (0.005 s/nt and 2.7 sequences respectively) samples (Fig 3B and 3D).

### Higher sequence diversity in the cervical tract compared to matched plasma samples early in infection

**Fig 4:**
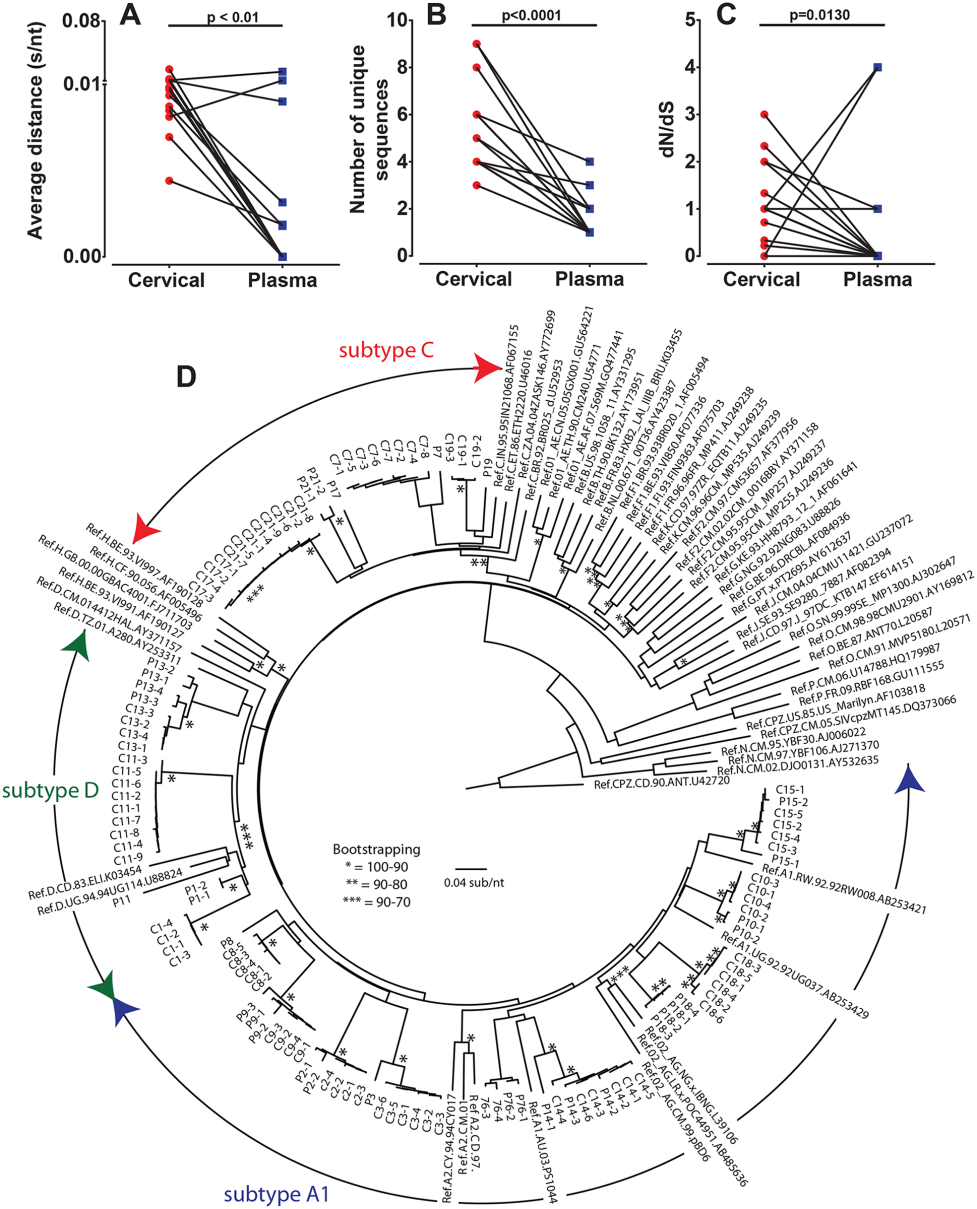
Envelope sequence diversity in the cervical tract and matched plasma samples early in infection. Paired cervical and plasma samples (n = 21) from individuals infected between 0–7 months were compared for C2-V3-C3 *env* diversity by calculating genetic distance using MEGA6 software (**A**). The number of unique sequences was derived by collapsing the number of total sequences (range = 93–2409 sequences) for each individual sample using online Galaxy software (**B**). Paired cervical and plasma sequences were analyzed for selective pressure by estimating the dN/dS ratio in each sample using SNAP v2.11 and plotted (**C**). Sequences of dS = 0 or dN = 0, resulting in dN/dS = 0 were included as zeros or no evolution. Statistical significance between matched cervical and plasma samples were determined using a two-tailed Wilcoxon matched-pairs signed rank test. The average of 100 maximum likelihood bootstrapped trees of nucleotide sequences were generated with MEGA6, rooted to the SIVcpz CD.90.ANT sequence, and visualized with FigTree 1.4.2 to highlight sequence heterogeneity. The C2-V3-C3 *env* sequences from cervical (C) and plasma (P) paired samples aligned to the reference HIV sequences from the Los Alamos Sequence Database (**D**) and following trees provide bootstrapping values.

In Fig 2, we compared total inter-patient genetic diversity and number of viral clones for paired and unpaired samples at very early and early infection. We now evaluate the intra-patient genetic diversity and number of viral clones with matched cervical and plasma samples from each available patient at the same sample collection time point (n = 15). Deep sequence analysis revealed higher envelope heterogeneity in cervical samples compared to that in the matched plasma from the same patient at the same time point (Fig 4A–4C). Matched cervical and plasma *env* sequences clustered together by patient on neighbor joining phylogenetic trees (Fig 4D) and show differences in the viral nucleotide (S1C and S1D Fig) and amino acid (S1A and S1B Fig) sequence diversity in plasma (S1B and S1D Fig) and cervical samples (S1A and S1C Fig). Average distances of 0.01 s/nt (range 0.004–0.02 s/nt) and 0.003 s/nt (range 0.0–0.02 s/nt) were found in matched cervical and plasma samples (Fig 4A), with the average number of unique sequences in cervical and in plasma samples being 5.4 clones (range 3–9 sequences) and 1.6 clones (range 1–4 sequences), respectively (Fig 4B). All of the cervical samples had greater number of distinct clones than the patient matched cervical samples. Only two showed higher genetic diversity in plasma compared to matched cervical samples. To ascertain if plasma samples underwent an immune selection process, dS/dN ratios of matched cervical and plasma samples were performed. A significantly lower ratio of dS/dN was seen in plasma samples compared to cervical indicating immune mediated deviations from the cervix to plasma (Fig 4C). However, it important to stress than in 10 of 15 plasma samples there was only one infecting clone, no genetic diversity, and as such no evolution to examine selective pressure.

In this matched patient/sample analysis, 81% of the 0–3 months cohort (Fiebig stage I-V) had higher genetic diversity in cervical samples compared to plasma (0.010 vs 0.0034 s/nt) (S7A Fig). All had a greater number of unique HIV-1 clones in the cervical (5.7; range 3–9) versus plasma samples (1.7; range 1–4) (S7C Fig). All 15 patients had more than one clone in their cervical samples whereas 5 plasma samples of the same patients had only a single HIV-1 clone. Similarly, the number of unique sequences in cervical samples was significantly higher (p<0.001) compared to the matched plasma samples during the very early 0–3 month phase of infection (S7A and S7C Fig), a finding not observed in cervical and plasma samples from early infection (3–7 months or Fiebig stage VI) (S7B and S7D Fig). No statistically significant differences could be determined when early time points were stratified for HIV-1 subtypes (S7E–S7H Fig), although there appears to be a reduced number of viral clones in plasma compared to cervical samples within individuals regardless of subtype at very early infection (S7G and S7H Fig).

Collectively, our cohort based analyses in Fig 2 and our matched patient/sample analyses in Fig 4 suggest that either (i) a genetic sieve may be in play to reduce the viral heterogeneity from the cervical mucosa to the systemic blood compartment or (ii) there is HIV evolution occurring in the cervical compartment that is absent in the plasma.

### Greater sequence diversity may be associated with faster CD4 T cell decline

**Fig 5:**
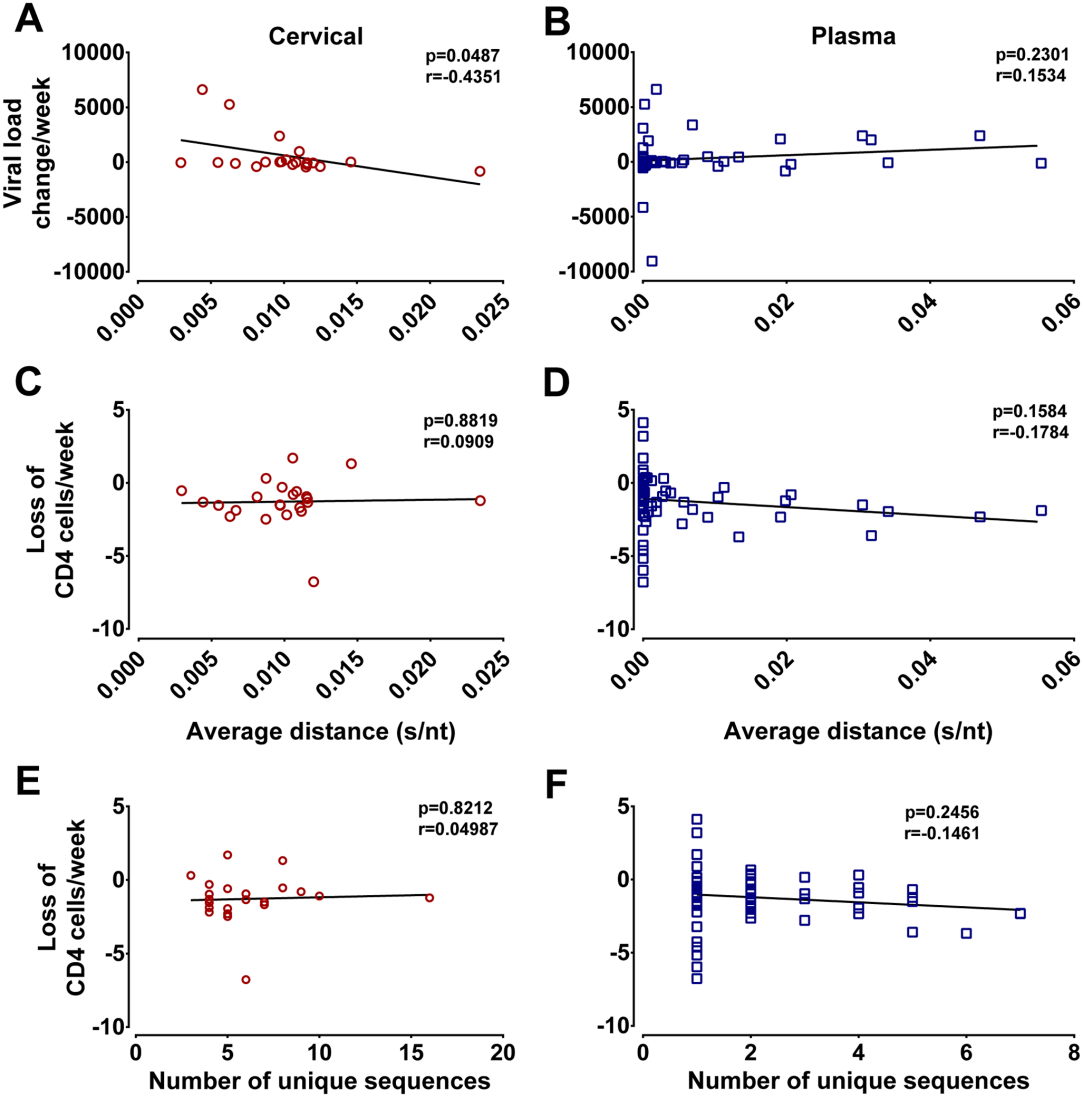
Sequence diversity in plasma influences CD4 T cell decline. Viral load and CD4 T cell slopes were plotted from up to 7 year longitudinal sampling of study subjects. Each slope was derived from a number of time points between viral set point to initiation of cART (S8 Fig). Spearman Rank correlation were calculated and presented for the average p-distance and viral load change for cervical (**A**) and plasma samples (**B**) and for the CD4 T cell loss compared to the HIV diversity in cervical (**C**) and plasma samples (**D**). Spearman Rank correlation were also calculated and presented for number of unique sequences versus CD4 T cell decline for cervical (p = 0.6463, r = 0.09446) (**E**) and plasma samples (0.0762, r = -0.2146) (**F**).

The study described herein is part of a longitudinal analysis of HIV disease progression in Ugandan and Zimbabwean women following very early/early infection and up to 9 years follow-up [27–29]. Combined antiretroviral treatment (cART) was provided when these women reached CD4 T cell counts of less than 200/mm^3^ of blood on two consecutive visits. We evaluated whether differences in viral diversity at very early/early infection influenced viral load kinetics (following viral set point to initiation of cART) and CD4 T cell counts during asymptomatic disease and up to initiation of cART (Fig 5). The *env* genetic diversity or *env* clonal number in the plasma or cervix at very early/early infection (Fig 2) was compared to the slopes of CD4 T cell declines and of viral RNA load increases in these patients (S8 Fig). While viral diversity at very early/early infection in cervical samples appeared to weakly correlate with the slope of viral load increases during subsequent disease (p = 0.0487, r = -0.4351) the same was not true for plasma samples (p = 0.2301, r = 0.1534) (Fig 5A and 5B). Of note, no correlation was observed between sequence diversity in cervical or plasma samples with the slope of CD4 T cell decline during subsequent disease (Fig 5C). In very early/early cervical and plasma samples, there was no correlation between the number of unique HIV sequences and the slope of CD4 T cell decline or viral load increases during disease (Fig 5E and 5F).

**Fig 6:**
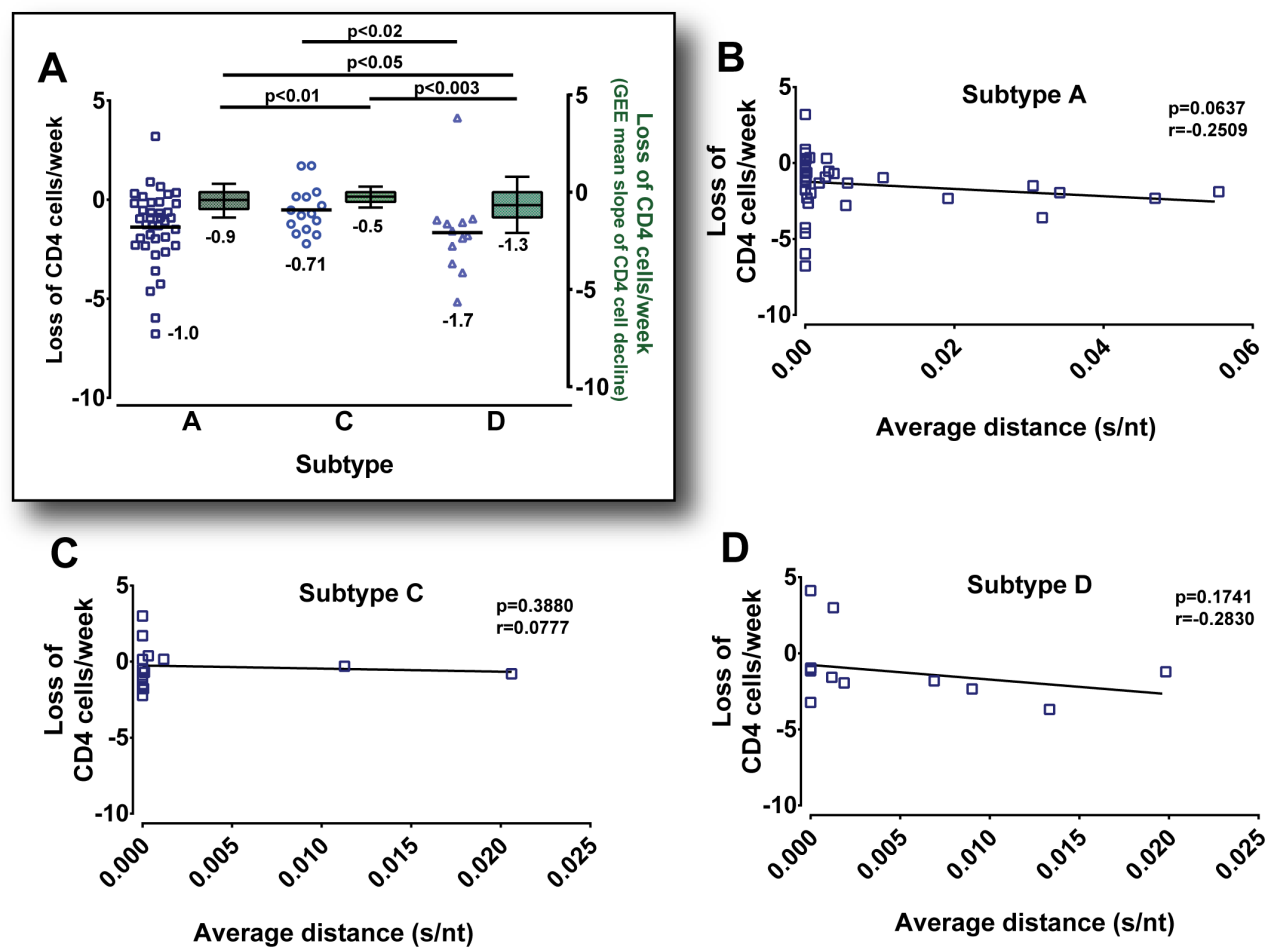
Slower CD4 T cell decline is associated with HIV-1 subtype infection. Declines in CD4 T cell rates per week from longitudinal sampling of study subjects (n = 72) were plotted. Each slope was derived from multiple time points of plasma sampling between viral set point and cART. Scatter dot plots of CD4 T cell declines in plasma stratified by subtype A, C and D infection are depicted on the left Y-axis (**A**). A marginal model with generalized estimating equation (GEE) approach was used to analyze CD4 T cell decline rates in a larger sample number of Ugandan and Zimbabwean women (n = 286) of the same cohort [24]. Box plots with mean and error bars of GEE calculated CD4 T cell declines grouped by subtype A, C and D are depicted on the right Y-axis (**A**). Statistical significance was done using a two-tailed Mann-Whitney test and two sample t-test assuming unequal variances. Spearman Rank correlation of the CD4 T cell loss versus average HIV genetic distance in the early plasma samples were separated and plotted by the infecting subtype A (**B**), subtype C (**C**) and subtype D (**D**).

Recently Venner *et al*., 2016 published data analyzing CD4 T cell declines in a total of 286 Ugandan and Zimbabwean women from this cohort. HIV-1 subtype C infection resulted in the slowest decline of CD4 T cells while HIV-1 subtype D infection caused the faster rate of CD4 T cell decline [24] (reproduced for Fig 6A). Thus we stratified our data by subtype A, C and D infection to remove confounding by subtype virulence and to access the influence of *env* genetic diversity in plasma on the slope of CD4 T cell declines during disease progression. In our study (subset of 80 of 286 patients), we observed a similar trend in the differential CD4 T cell decline based on HIV subtype infection, e.g. subtype C infections had the slowest loss in CD4 T cells (Fig 6A). In this subset analyses, subtype D infections progressed more rapidly to AIDS than subtype C HIV-1 infections (p<0.02), in keeping with that observed in the full cohort (p<0.003).

Previous studies have suggested that higher diversity of infecting HIV (in plasma) may result in faster disease progression (Sagar M J Virol 2003). However, to make this comparison in our cohort, analyses had to be compared by subtype. We did not observe a correlation between HIV diversity at early infection (in plasma or endocervix) on subsequent disease progression (Fig 6B, 6C and 6D). However, with the HIV-1 subtype A group there was a trend for increased disease progression (based on the rate of CD4 decline) in those women infected with a more diverse HIV-1 population. We only note this trend because the previous study (Sagar M J Virol 2003) was largely focused on HIV subtype A infections in Kenya.

## Discussion

In this manuscript we provide *in vivo* evidence for the existence of a stringent bottleneck between the cervical mucosa and the systemic compartment based on the reduced *env* diversity present in plasma when compared to viruses isolated from the mucosa early in infection. It is widely understood that a stringent transmission bottleneck occurs during heterosexual HIV-1 infection. This leads to the establishment of a systemic infection by a single viral founder in 76–90% of the cases [1, 12, 13, 30] despite the likely exposure to a diverse HIV-1 population from the donor via semen or blood. The exact barrier site(s) in the donor and/or recipient as well as factors impacting this genetic bottleneck are poorly understood. It is important to also stress that the samples for this study were collected at least one month post infection/exposure. Thus, the infecting HIV may have evolved in the female genital tract but by this logic, it is unclear why similar evolution has not occurred in the blood, where one HIV-1 clone appears to persist. In more later (3–7 months) versus early (0–3 months) primary infection, there are fewer patients that harbor only a single infecting HIV strain in plasma (40% at 3–7 months versus 66% at 0–3 months). However, we failed to observe a correlation between the length of infection prior to sampling and the HIV genetic diversity of that cervical or plasma sample. In addition there was no evidence of super/coinfection and no correlation between HIV diversity in plasma or endocervix with the number of sexual partner or coital events around the time of transmission. We propose that with infection of the female genital tract, this may be related to the differential HIV diversity of the donor inoculation leading to infection. Unlike the historical spread of HIV in the MSM population in high income countries attributable to acute/early to acute/early transmission in donor/recipient pairs, this cohort of women of child bearing age and seeking treatment at family planning clinics were more likely exposed and infected by known set of potential male donors through heterosexual contact.

In this study, we had the rare opportunity to examine both the blood and genital compartment for HIV genetic composition soon after heterosexual male-to-female infection in Uganda and Zimbabwe [27–29, 31]. These women were then monitored for disease progression for an average of 5 years (3–9 years range) prior to initiating cART when their CD4 cell counts dropped below 200/mm^3^ of blood. In this report, we clearly show that at early infection (0 to 90 days) the cervical compartment contained a diverse set of HIV-1 clones while the blood compartment had a median of one HIV clone—consistent with previous reports [1–3]. The higher HIV genetic diversity in the genital tract compared to the blood compartment at early infection was not associated with hormonal contraceptive use, age, past pregnancies, or the number of coital events or sex partners during the 3 months preceding early sample analyses. Y chromosome or PSA antigen was not detected in any of the cervical swabs obtained at acute infection (Fiebig stage I), very early (Fiebig stage II to V) or at early infection (Fiebig stage V), i.e. the same samples employed for HIV-1 genetic diversity analyses. X chromosome was detected in a subset of endocervical samples. These findings indicate that a stringent genetic bottleneck occurs between the genital tract and blood of women during primary infection.

Exactly how HIV-1 enters the body through the vaginal mucosal surfaces is an active area of investigation, with epithelial breaches, tissue trauma, transcytosis and cell-associated transport all acting as potential portals of entry. The HIV genetic bottleneck may be simply due to stochastic events and chance infection of susceptible cells which leads to expansion and subsequent systemic infection. SIV studies in macaques suggest that CCR6+ CD4+ Th17 cells are preferential targets for early infection in the vaginal mucosa, possibly due to high levels of CCR5 expression [32]. The low density of Th17 cells in the vaginal mucosal tissue may result in a low frequency of contact between inoculating virus and susceptible cells resulting in very few foci of infection and thus, resulting in the genetic bottleneck. HIV-1 isolates obtained from acute infection compared to chronic HIV-1 strains, aside from requirement for CCR5 usage, do not appear to have any preferential tropism or enhanced replication kinetic in Th17 cell lines let alone in CD4+CCR5+ primary T cell, T cell lines, monocyte-derived macrophages, or dendritic cell-T cell co-cultures. Based on this study and previous reports, it appears unlikely that successful male-to-female HIV transmission is related to a very low frequency infection of Th17 cells in vaginal mucosa and subsequent systemic infection. First, in the presence or absence of systemic infection of macaques, multiple foci of SIV infection were observed in the vaginal mucosa by in situ staining when macaques were exposed to vaginal SIV inocula, albeit at very high titer [33, 34]. Second, in our current study, swab collection from the endocervix yielded multiple genetically distinct clones of HIV.

Unlike the blood, the endocervical mucosa is a static tissue and we sampled less than 1/1000^th^ of the overall female genital tract surface suggesting that other distinct HIV-1 clones may have been replicating in different foci across the vaginal vault. We also sampled a very small fraction of the plasma but in this case, HIV is more thoroughly mixed in plasma fluid. Nonetheless, HIV likely migrates to draining lymph nodes during early infection [35]. Plasma HIV may or may not be a true representation compartmentalized HIV-1 population in the lymph node. Interestingly, the single HIV-1 clone found in the blood was rarely the dominant clone found in the cervical HIV-1 population sampled by our swab at the same time point. These findings would suggest that the vaginal tract contains even higher HIV-1 heterogeneity than what we sampled in the endocervix. We are extrapolating homogeneity and diversity of the infecting HIV based on only the C2-V3-C3 region of env sequence, which is only 1/40^th^ of the full genome length. Although we would fully anticipate some sequence variation in the remainder of the genome, especially in the plasma HIV from later Fiebig stage V, the hypervariable V3 region still remains the one of the best proxy for relative HIV evolution across the genome. In 27% of early infections, we did observe more than one HIV clone in the plasma at early infection. Again, there was no correlation between the appearance of multiple HIV clone with the timing of sample collection, number of sexual partners, sexual acts, and other at risk behavior near the time of transmission.

**Fig 7:**
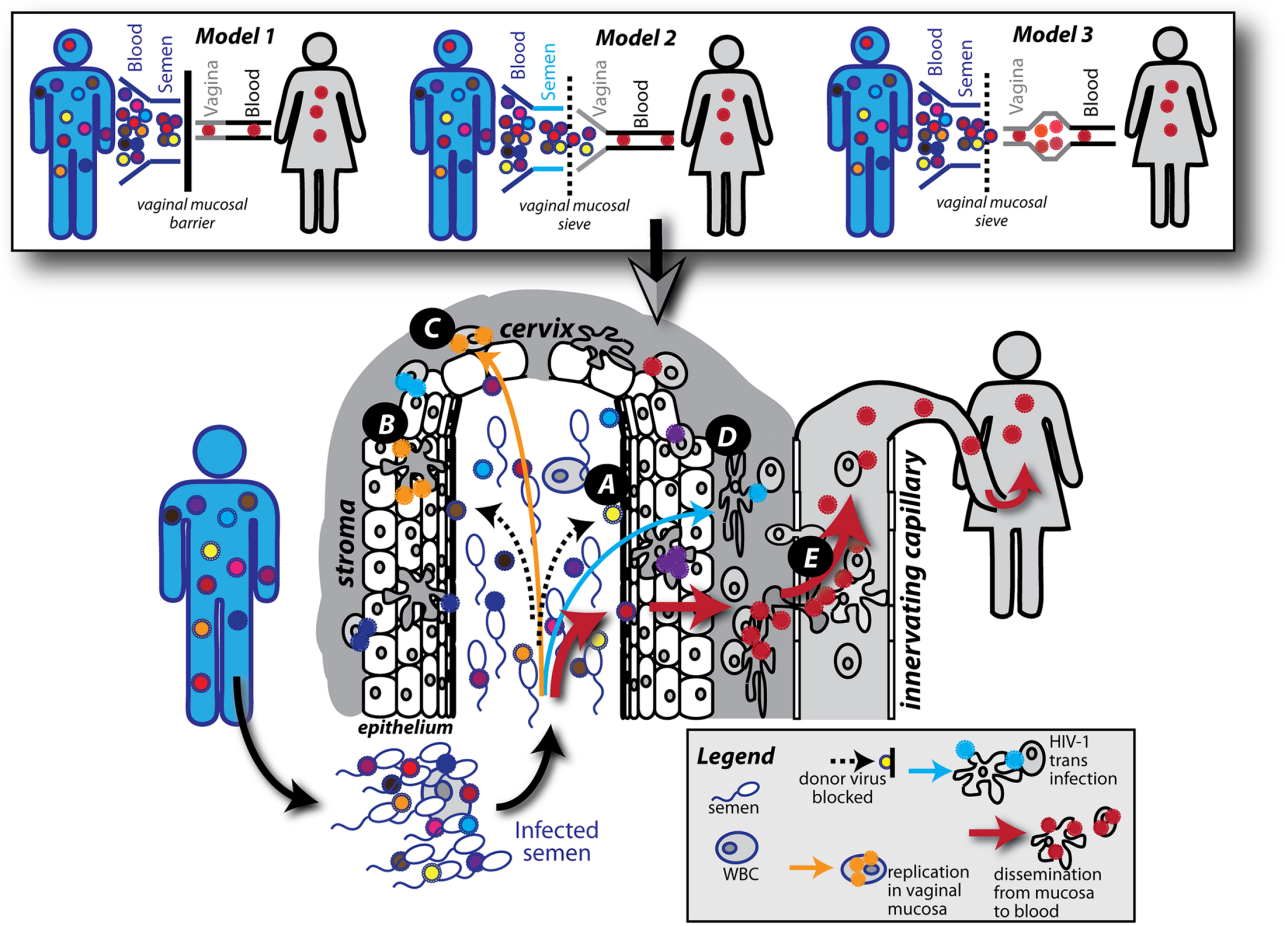
Models of heterosexual HIV-1 transmission bottlenecks. Donors infected with HIV-1 have a genetically diverse HIV-1 population in the blood. Within the donor, a genetic bottleneck has been reported to occur between the blood compartment and semen, resulting in less diverse HIV-1 populations. The intact female vaginal mucosa is known to act as an efficient barrier to HIV-1 transmission, resulting in only a single TF variant establishing infection in the recipient (**Model 1**). Aside from the bottleneck within the donor the most stringent HIV-1 sieve effect is thought to take place in the female genital tract. In this study we show high HIV genetic diversity and distinct HIV-1 clones in the endocervix during early infection suggesting either an infection of the female genital tract with a population of HIV-1 clones from the donor (**Model 2**) or infection of the female genital tract with a single or limited number of HIV-1 clones which then evolves prior to systemic transmission (**Model 3**). Model 2 has been expanded in the bottom schematic figure. Transmission processes across the female genital tract: HIV-1 virions from the donor fluid are trapped by mucus or entry is blocked by the physical barrier of the mucosae (**A**). HIV-1 virions can be captured and internalized by intraepithelial Langerhans cells (**B**). HIV-1 can infect stromal CD4 T cells leading to productive infection (**C**). HIV-1 virions can bind to and infect DCs in the stroma and infect CD4 T cells *in trans* through an infectious synapse (**D**). Infected CD4 T cells and DCs can disseminate the virus from the mucosa to the blood (**E**).

Our findings suggest that infection of the blood from the vaginal tract may represent an additional genetic bottleneck during transmission (**Model 2** Fig 7). However, it is important to note that we could not access the previously described HIV transmission bottleneck from donor blood to donor semen, or from donor semen to recipient vaginal tract (**Model 1** Fig 7). Finally, we must also propose that HIV may evolve faster in female genital tract than in the blood. This may relate to more intensive selective pressure by the innate and then acquired immune response in the female genital tract than observed systemically (**Model 3** Fig 7). It may also relate to strong bottleneck during transmission to the female genital tract followed by HIV evolution in this compartment and then bottleneck for transmission to the blood. It is possible that all three models exist simultaneously. Certainly, higher viral loads in the donor lead to higher probability of transmission but it remains unclear if higher levels of HIV in semen, deposited into the vaginal vault would lead to infection by more than one HIV-1 variant in the blood. In our study, the donors were not identified and no blood or semen was available. For models 2 and 3, transmission of only one HIV-1 clone across the vaginal mucosal barrier to the blood could still be due to purely stochastic events. Several studies suggest that the composition of the mucosal barrier may impact both the probability of transmission as well as the HIV heterogeneity in the blood during acute infection [36–40]. A minimal physical barrier is obvious in direct blood-to-blood transmission and yet there is a weak genetic bottleneck, albeit less than that observed with male-to-female HIV-1 transmission during coitus. There are suggestions that transmission of more HIV-1 clones to the blood during anal receptive transmission as compared to that resulting from coitus is related to a weaker gut mucosal barrier. However, the physical act of anal receptive sex versus vaginal sex generally results in more tissue damage due to bruising and lacerations which may contribute more to the infecting HIV having greater heterogeneity. Interestingly, four of these 65 women participants had two very distinct and genetically diverse HIV-1 clones in the blood, which could be chance or relate to abnormal damage to mucosal barrier during coitus. However, factors that may impact the probability of transmission, i.e. number of sex partners, of coital acts, and lack of condom usage for 3 months preceding seroconversion, did not impact the heterogeneity of the HIV-1 in the cervix or in the blood. Several studies have now shown a slight but significantly increased risk of HIV acquisition with the injection of depot-medroxyporgesterone acetate (DMPA) for birth control [31, 41–43]. In macaques, treatment with DP thins the vaginal lining and increases the probability of SIV infection [43, 44]. However, there was no evidence that combined oral contraceptives or DP use had any impact on the heterogeneity of HIV infecting the cervix or blood. Again, in all of the latter conditions, the number of distinct HIV-1 clones in the cervix (median of 5.7) was 3.3-fold higher than in the blood (median of 1.7).

There is mounting evidence that specific genotypic and phenotypic characteristics on the infecting HIV-1 clone may impact subsequent disease progression. For example, a lower replicative fitness of subtype C versus subtype A or D HIV-1 isolates at acute/early infection correlated with lower disease progression in this cohort [24]. Also, lower replication capacity of HIV-1 at acute/early infection was also observed in elite suppressors regardless if specific protective HLA alleles (e.g. B27 and B57) were present or absent [45, 46]. In regards to genotype, an early study analyzing HIV-1 heterogeneity at acute/early infection described faster disease progression in patients infected with more diverse HIV clones [47]. However, follow up studies could not reproduce these findings but these analyses were often confounded by shorter longitudinal follow ups and varying times in cART initiation. In our study, all HIV-infected women were followed for a range of 3–9 years from acute/early infection to the same endpoint, the initiation of cART when CD4 cell counts dropped below 200/mm^3^ on two consecutive visits. The genetic diversity and number of distinct clones in the cervix showed no relationship to disease progression. However, greater HIV-1 heterogeneity in the blood at very early/early infection was related to faster declines in CD4 cell counts, which appears to confirm the Sagar *et al*., 2003 study [47].

In summary, we provide compelling *in vivo* evidence of the existence of the intra-patient genetic bottleneck occurring at the cervical mucosae during the earliest stages of HIV-1 heterosexual infection. We can demonstrate that a reduced number of viral clones with lower *env* diversity exist in plasma compared to the mucosal compartment when analyzing the HIV-1 C2-V3-C3 *env* diversity of a total of 28 endocervical swab and 67 plasma samples, and subsequently stratifying them into very early (0–3 months) and early (3–7 months) infection from one of the largest natural cohorts of African women. The data presented here will have significant implications in rational vaccine design where a profound understanding of the early events of viral transmission, restriction and dissemination are important factors. It is widely accepted that harnessing anti-HIV humoral and cellular immune responses are required to prevent or abort infection at the earliest time points. Identifying and characterizing the diversity of HIV-1 in the donor and recipient compartments involved in transmission may provide better applications of HIV-1 preventative therapies. These studies will allow to more accurately and efficaciously target the virus at the most susceptible states. Furthermore, the existence of multiple viral clones with increased diversity in the mucosa will have a significant impact on the design of treatment and eradication/cure strategies.

## Materials and methods

### Study participants

Samples were obtained from HIV-1 infected Ugandan and Zimbabwean women from a well described cohort [31]. Women were enrolled into the “Hormonal Contraception and HIV-1 Genital Shedding and Disease Progression among Women with Primary HIV Infection (GS) Study” [27–29], after they were diagnosed for HIV-1 infection while participating in the “Hormonal Contraception and Risk of HIV Acquisition Study” in Uganda and Zimbabwe [31]. Serum samples were previously tested for HIV by enzyme linked immunosorbent assays (ELISA) [Recombigen HIV-1/HIV-2 (Cambridge Biotech, Galway, Ireland), Organon Vironostika (Organon Teknika, Durham, North Carolina, USA), Abbott Murex (Abbott Park, Illinois, USA), Sanofi (Sanofi Diagnostics Pasteur, Redmond, Washington, USA)] and confirmed with rapid testing [HIV SAV1 or SAV2 (Savyon Diagnostics, Ashdod, Israel), Capillus HIV-1/HIV-2 (Trinity Biotech USA, Jamestown, New York, USA) or Determine (Abbott)] and Western blot (BioRad, Hercules, California, USA) or PCR (Amplicor HIV-1 DNA test, version 1.5, Roche Diagnostics, Branchburg, New Jersey, USA). For a confirmed serum positive HIV-1 test, HIV PCR was performed on previous visit samples. The time point of initial infection was estimated as midpoint between the last negative and first positive HIV-1 test. The estimated infection date will fall within a six week window of the actual infection date as testing was performed every 3 months [31]. Women were followed up to 9.5 years, and blood and endocervical swab samples were collected every 3 months. Plasma and cervical sample viral loads as well as plasma CD4 T cell counts were measured in almost every sample. Combination Antiretroviral Therapy (cART) and trimethoprimsulfamethoxazole (for prophylaxis against bacterial infections) were offered to study subjects if they developed severe symptoms of HIV infection or who had CD4 T cell counts equal to or less than 200 cells/mm^3^ on two consecutive visits. For the study described here, women were chosen from the cohort if they were enrolled within 7 months of infection. Among the 72 selected women, 49 were enrolled within 3 months and 23 between 3 and 7 months of infection (S1 Table).

### Ethics statement

Written informed consent was provided by all study participants. Ethical approval was obtained from the Institutional Review boards (IRBs) from the Joint Clinical Research Centre and the Ugandan National Council for Science and Technology (UNCST) in Uganda, from the University of Zimbabwe, from the University Hospitals of Cleveland (02-01-09 and 07-02-32) and from Western University (105737). All investigations have been conducted according to the principles expressed in the Declaration of Helsinki.

### Sample collection

To collect endocervical samples a dacron-dipped swab was inserted and rotated on the cervical outer surface for 3–5s and subsequently placed in RNAlater (Invitrogen, USA). Virus was pelleted by ultracentrifugation using a Biofuge rotor at 23,000 rpm for one hour. The virus pellet was resuspended and viral RNA levels were evaluated using a Roche Amplicor HIV-1 Monitor Test, version 1.5 with a detection limit of 50 RNA copies/ml (Roche, CHE). Plasma viral loads were measured using standard procedures of the same Roche test as described. Cervical and plasma viral loads were analyzed in a Virology Quality Assurance (VQA) certified laboratory in Kampala, Uganda [29]. CD4 T-lymphocyte counts were determined by standard 4-color flow cytometry using FACSCalibur (Becton Dickinson, USA).

### PCR amplification

RNA was extracted from plasma and cervical samples using a viral RNA mini kit (Qiagen, DE). cDNA synthesis was performed using Moloney Murine Leukemia Virus (MMLV) (Invitrogen, USA) and primer ENVM 5’-TAGCCCTTCCAGTCCCCCCTTTTCTTTTA-3’ (HXB2 9068–9096). The C2-V3-C3 region of envelope was amplified by an external-nested PCR amplification using the primers forward ENVB 5’-AGAAAGAGCAGAAGACAGTGGCAATGA-3’ (HXB2 6202–6228) and reverse ED14 5’- TCTTGCCTGGAGCTGCTTGATGCCCCAGAC-3’ (HXB2 7932–7961) (external) and forward E80 5’-CCAATTCCCATACATTATTGTG-3’ (HXB2 6858–6879) and reverse E125 5’-CAATTTCTGGGTCCCCTCCTGAGG-3’ (HXB2 7315–7338) (nested) [48]. PCR conditions were as followed: 0.2 μM of each primer, 1.5 mM MgCl2, 1x Platinum Taq PCR buffer, 0.2 mM dNTPs and 2 units Platinum Taq DNA polymerase. PCR cycle conditions were 95°C for 2min, followed by 35 cycles of 95°C for 30s, 55°C for 30s and 72°C for 2min (external) or 45s nested, and a final extension of 72°C for 10min. To prepare the amplicon library for 454 sequencing, fusion primers including the Roche 454 titanium key sequence, a multiplex identifier (MID) sequence for forward and reverse primers followed by the template specific sequence were generated. The sequence specific primers were: forward (E110) 5’-CTGTTAAATGGCAGTCTAGCAGAA-3’ (HXB2 7002–7025) and reverse (E125) 5’-CAATTTCTGGGTCCCCTCCTGAGG-3’ (HXB2 7315–7338). PCR cycle conditions were 95°C for 2min, followed by 35 cycles of 95°C for 30s, 55°C for 30s and 72°C for 45s, and a final extension of 72°C for 10min. The PCR products were run on a 1% agarose gel at 100V for 45min to verify products of 406 bp. Confirmatory sequencing on a small subset of samples, out of a greater ongoing diversity study was performed using the Illumina MiSeq platform.

### Sample library preparation and NGS

All PCR products were purified using the Agencourt AMPure XP bead system with a bead:DNA ratio of 0.7:1 according to the Roche manual. Following purification the sample libraries were quantified using the Quant-iT PicoGreen ds DNA assay kit (Invitrogen, CA) according to the Roche 454 library preparation instructions. Quantified samples were diluted and pooled together at 10^6 molecules/μl for pyrosequencing. For emulsion PCR (emPCR) a ratio of 0.5 molecules of sample library per bead was chosen. EmPCR was performed according to the Roche 454 manual. Following emPCR and DNA library enrichment, 500,000 enriched beads were loaded onto the titanium picotiter plate according to the Roche instructions. The sequencing run was performed on the Roche 454 GS Junior sequencer using the 200 nucleotide cycles (reads up to 500 bases) and full processing for amplicon libraries settings.

### Sequence data analysis

Raw sequence data were extracted by the MID tag using a custom analysis pipeline. The 454 amplicon adapters were trimmed and sequences of less than 200bp were discarded. Sequences were edited using BioEdit v7.2.5 and a multiple sequence alignment of these data including HIV-1 subtype A, B, C and D reference sequences curated by the Los Alamos National Laboratory HIV Sequence Database (http://www.hiv.lanl.gov) was generated using MUSCLE [49, 50]. Neighbor joining and maximum likelihood trees were constructed with SEAVIEW 4 [51] and visualized with FigTree 1.4.2. Genetic distance analysis within each sample were conducted using MEGA 6 [52] and is expressed as substitutions per nucleotide (s/nt). The number of unique sequences was identified by collapsing the total number of sequences (ranging from 900–1500 sequences) using Galaxy [53, 54]. To identify the unique sequences, the multiplicity values were used to calculate the 1% cut off for the total number of input sequences for each sample. The resulting sequences were then defined as “unique sequences”. Error rates from PCR and 454 sequencing processes have been calculated to be between 0.1% and 0.6% [15, 55–58]. Therefore, we decided to use a cut off of 1% that is higher than the calculated error rates. For this reason, unique clones were defined as sequences that had a higher frequency than 1% of the total number of input sequences. Selective pressure were analyzed using the Los Alamos National Laboratory Synonymous Nonsynonymous Analysis Program (SNAP v2.11) (http://www.hiv.lanl.gov) to estimate the number of synonymous (dS) and nonsynonymous (dN) sites in the sequence alignments, and synonymous and nonsynonymous base substitutions (dS/dN) in each sample [59]. Sequences that lacked a synonymous (dS = 0) or nonsynonymous (dN = 0) substitution were not included in the calculation. All highlighter plots were generated using lanl Highlighter tool (http://www.hiv.lanl.gov) [1].

### X-Y chromosome PCR of endocervical swab samples

PCR amplification of the ZFX and ZFY gene located on the X and Y chromosome respectively was performed using a shared forward primer ZFX/ZFY_F: 5’-ATTTGTT CTAAGTCGCCATATTCTCT-3’ and a X- chromosome specific ZFX_R: 5’-GAACACACTACTGAGCAAAATGTATA-3’ or Y- chromosome specific ZFY_R: 5’-CATCTTTACAAGCTTGTAGACACACT-3’ reverse primer. PCR cycle conditions were 94°C for 2min, followed by 35 cycles of 94°C for 60s, 57°C for 30s and 72°C for 60s and a final extension of 72°C for 10min. PCR products were run on a 1% agarose gel at 100V for 30min.

### Assessment of viral load increase and CD4 T cell decline

Blood samples were taken at regular intervals up to 7 years from HIV-1 diagnosis and viral loads and CD4 T cell counts were assessed. The slope of the viral RNA load increase and the slope of the CD4 T cell decline over the infection period was analyzed by linear regression calculation using GraphPad Prism 6. Each slope was derived from a number of time points chosen between viral set point to initiation of cART. At least 3 time points and up to 14 were used for the analysis dependent on the disease progression. Study subjects received cART if CD4 T cell counts were equal to or less than 200 cells/mm^3^ on two consecutive visits or if they developed severe symptoms of HIV infection, which happened between 1 and 7 years post infection.

## Supporting information

S1 Table Summary of volunteer infection status and sample collection date. Sample IDs highlighted in bold-italic represent matched endocervical and plasma sample pairs. # Estimated time point of initial infection. The date of infection was estimated as midpoint between the date of the last negative and first positive HIV-1 test. DPI: days post infection. DPI are calculated as number of days between Sample Date and the estimated date of infection (Start Date).
(PDF)

S1 Fig Visualization of nucleotide and amino acid sequence diversity in cervical and plasma samples from early infection and in plasma samples from chronic disease. Paired cervical and plasma consensus sequences spanning the C2-C3 region of *env* for the 15 individuals are presented in highlighter plots with the dominant sequence in each patient sample/compartment utilized as consensus. The nucleotide highlighter plot for the paired cervical and plasma samples are presented in panels **A** and **B**; amino acid highlighter plots in panels **C** and **D**, respectively. Nucleotide base changes (or amino acid substitutions) are highlighted in color code shown in the legend. The thickness of each line in the highlighter plot provides the amount of sequence reads as described in the legend. Panel **E** provides the nucleotide alignment highlighter plots for the additional HIV sequences from unpaired cervical samples from early infection whereas panels **F** and **G** display those nucleotide alignment highlighter plots for the additional unpaired plasma sample from early infection. Nucleotide (**H**) and amino acid (**I**) highlighter plots are also presented for the shorter V3 sequences from plasma samples from eight chronically infected, untreated patients from a Spanish cohort [15].
(PDF)

S2 Fig Transition and transversion mutations and the synonymous versus nonsynonymous nucleotide substitution ratio (dS/dN) in cervical and plasma *env* sequences from early and chronic infection. As described the percentage of A-to-G/G-to-A, C-to-T/T-to-C (transition mutations), A-to-T/T-to-A, C-to-G/G-to-C, G-to-T/T-to-G, and A-to-C/C-to-A (transversion mutations) were plotted (**A**) for the intrapatient HIV quasispecies in the early cervical and plasma samples as well as in the plasma samples from chronically infected patient [15]. Cervical and plasma sequences were analyzed for selective pressure by estimating the dN/dS ratio in each sample using SNAP v2.11 (**B**). Sequences of dS = 0 or dN = 0, resulting in dN/dS = 0 were excluded from the analysis under the label “Plasma with diversity”. Positive selection is evident in samples where dN/dS ratios are greater than 1. All substitutions were determined from the dominant HIV clone in each patient sample. Statistical significance were determined using ANOVA.
(PDF)

S3 Fig Phylogenetic trees of the HIV-1 C2-C3 sequences derived from the endocervical (A) and plasma (B) samples at early infection. The average of 100 maximum likelihood bootstrapped trees of nucleotide sequences were generated with MEGA6, rooted to the SIVcpz CD.90.ANT sequence, and visualized with FigTree 1.4.2 to highlight sequence heterogeneity. The HIV-1 C2-V3-C3 *env* sequences from cervical (**A**) and plasma (**B**) samples aligned to the reference HIV sequences from the Los Alamos Sequence Database.
(PDF)

S4 Fig Clinical predictors on *env* sequence diversity in cervical and plasma samples do not influence viral diversity. Women enrolled in the prior hormonal contraceptive study were administered combined oral contraception (COC), Depot-medroxyprogesterone acetate (DP) or did not receive any hormonal contraception (NH). Upon HIV diagnosis the women were transferred into the current study and their viral diversity in plasma and cervical mucosa evaluated based on the contraception protocol they utilized (**A**). The incidence of pregnancies (**B**), the number of sexual partners (**C**), concurrent sex acts (**D**), incidence of new sexual partners (**E**), the primary partner risk behavior (including the partner being HIV+, abnormal discharge from penis, weight loss, if the partner had sex with another woman or partner spends nights away from home) (**F**) and participant behavior risk (including having multiple partners, a new sex partner, is engaged in commercial sex work or had sex with another man in the last 3 months) (**G**) were also stratified based on HIV viral diversity. Statistical analysis was done using a two-tailed Mann-Whitney test.
(PDF)

S5 Fig Effect of clinical predictors on number of unique sequences found in cervical and plasma samples. The number of unique sequences identified in cervical and plasma samples were analyzed based on clinical predictors. These included the use or absence of hormonal contraception (**A**), the incidence of pregnancies (**B**), the number of sexual partners (**C**), concurrent sex acts (**D**), incidence of new sexual partners (**E**), the primary partner risk behavior (including the partner being HIV+, abnormal discharge from penis, weight loss, if the partner had sex with another woman or partner spends nights away from home) (**F**) and participant behavior risk (including having multiple partners, new sex partner, engaged in commercial sex work or had sex with another man in the last 3 months) (**G**). Statistical analysis was done using a two-tailed Mann-Whitney test.
(PDF)

S6 Fig Sexual activity and the use of condoms does not influence HIV-1 *env* genetic diversity and number of clones in cervical and plasma samples. Predictors of the number of sex acts (**A-D**) and the number of sex acts where participants were using a condom (**E-F**) were evaluated based on the average genetic diversity of HIV (s/nt) in the cervical (**A** and **E**) and in the plasma (**B** and **D**) compartments or based on the number of unique HIV sequences in the cervical (**C** and **G**) and in the plasma (**D** and **H**) compartments. Each symbol represents an individual patient. Statistical significance was performed using a Spearman Rank correlation analyses.
(PDF)

S7 Fig C2-V3 envelope sequence diversity in paired cervical and plasma samples during very early and early in infection. Genetic distance and number of unique sequences of paired cervical and plasma samples were grouped into very early (0–3 months, n = 14) and early infection (3–7 months, n = 7) and separated by HIV-1 subtype of infection (A, C and D) (**A-H**). Samples were separated by subtype A, C or D and average distance and number of unique sequences plotted as described previously (**E-H**). Statistical analysis for significance between cervical and plasma samples were determined using a two-tailed Wilcoxon matched-pairs signed rank test (exact p value is shown; N.S., not significant).
(PDF)

S8 Fig Longitudinal analysis of viral loads and CD4 T cell counts. Plasma viral loads were assessed from multiple time points up to 7 years from HIV diagnosis and stratified according to linked cervical (**A**) or plasma samples (**B**). Likewise, the CD4 T cell counts were also assessed according to linked cervical (**C**) or plasma samples (**D**). Plasma viral loads were determined using a Roche Amplicor HIV-1 Monitor Test, version 1.5 while CD4 T cell counts were determined using an optimized 4-colour flow panel on a FACSCalibur flow cytometer. Linear regression analysis was performed using GraphPad PRISM 6 version 6.07.
(PDF)
