# Supplementary material for: Higher sequence diversity in the vaginal tract than in blood at early HIV-1 infection
Source: PLoS Pathog. 2018 Jan 18;14(1):e1006754. doi: 10.1371/journal.ppat.1006754 (PMC5773221; doi:10.1371/journal.ppat.1006754)
Supplement: S1 Table — Sample IDs highlighted in bold-italic represent matched endocervical and plasma sample pairs. # Estimated time point of initial infection. The date of infection was estimated as midpoint between the date of the last negative and first positive HIV-1 test. DPI: days post infection. DPI are calculated as number of days between Sample Date and the estimated date of infection (Start Date). (PDF) [file ppat.1006754.s001.pdf]

S1 Table: Summary of volunteer infection status and sample collection date.

Sample	Start Date#	Sample Date	DPI	Fiebig stage
plasma	20-Dec-2001	31-Jan-2002	41	V
plasma	23-Oct-2000	03-Apr-2001	160	VI
plasma	07-Mar-2001	06-Apr-2001	29	V
plasma	17-Nov-2000	20-Apr-2001	153	VI
plasma	27-Nov-2001	20-Feb-2002	83	V
plasma	19-Oct-2001	26-Feb-2002	127	VI
plasma	27-Mar-2001	06-Jun-2001	69	V
plasma	31-May-2001	27-Jun-2001	27	IV
plasma	20-Dec-2002	04-Feb-2003	44	V
plasma	10-Dec-2001	03-Apr-2002	113	VI
plasma	06-Aug-2001	29-Aug-2001	23	III
plasma	17-Dec-2003	13-Feb-2004	56	V
plasma	13-Jan-2003	21-Feb-2003	38	V
plasma	29-Jan-2007	29-Jan-2007	0	I
plasma	22-Apr-2002	30-Apr-2002	8	I
plasma	14-Jun-2001	25-Sep-2001	101	VI
plasma	12-Apr-2002	03-May-2002	21	III
plasma	13-Feb-2003	04-Mar-2003	21	III
plasma	30-Jan-2002	14-May-2002	104	VI
plasma	01-Aug-2001	12-Nov-2001	101	VI
plasma	12-Jan-2006	14-Feb-2006	32	V
plasma	13-Nov-2003	19-Mar-2004	126	VI
plasma	03-Mar-2004	05-Apr-2004	32	V
plasma	16-Jan-2007	23-Feb-2007	37	V
plasma	05-Jul-2002	02-Aug-2002	27	IV
plasma	15-Aug-2002	03-Sep-2002	18	II
plasma	29-Aug-2002	17-Sep-2002	18	II
plasma	18-Sep-2002	11-Oct-2002	23	III
plasma	28-Aug-2002	05-Nov-2002	67	IV
plasma	28-Oct-2002	26-Nov-2002	0	I
plasma	23-Jun-2004	20-Jul-2004	27	IV
plasma	30-Jun-2004	21-Jul-2004	21	III

Sample ID	Sample	Start Date#	Sample Date	DPI	Fiebig stage
GS-0189-05	plasma	21-Jan-2005	22-Jul-2005	181	VI
GS-0201-05	plasma	18-Apr-2005	29-Jul-2005	101	VI
GS-0202-05	plasma	04-Feb-2005	29-Jul-2005	175	VI
GS-0205-05	plasma	05-Apr-2005	29-Jul-2005	114	VI
GS-0211-07	plasma	06-Jul-2007	06-Jul-2007	0	I
GS-0216-03	plasma	09-Sep-2003	07-Oct-2003	28	IV
GS-0216-04	plasma	08-Jul-2004	16-Aug-2004	38	V
GS-0221-03	plasma	16-Sep-2003	13-Oct-2003	27	IV
GS-0243-05	plasma	02-Aug-2005	13-Sep-2005	41	V
GS-0247-04	plasma	17-Aug-2004	14-Sep-2004	27	IV
GS-0265-04	plasma	16-Jun-2004	29-Sep-2004	103	VI
GS-0296-04	plasma	16-Sep-2004	29-Oct-2004	43	V
GS-0308-04	plasma	12-May-2004	09-Nov-2004	177	VI
GS-0310-05	plasma	07-Jun-2005	04-Nov-2005	147	VI
GS-0323-04	plasma	19-Aug-2004	22-Nov-2004	93	VI
GS-0387-06	plasma	07-Nov-2006	07-Nov-2006	0	I
GS-0388-06	plasma	07-Nov-2006	07-Nov-2006	0	I
GS-0411-06	plasma	27-Nov-2006	27-Nov-2006	0	I
GS2003	plasma	01-Sep-2000	19-Mar-2001	198	VI
GS2005	plasma	27-Jan-2001	20-Mar-2001	53	V
GS2007	plasma	01-Nov-2000	28-Mar-2001	147	VI
GS2012	plasma	02-Nov-2000	04-Apr-2001	152	VI
GS2038	plasma	18-May-2001	25-Jun-2001	37	V
GS2047	plasma	05-Apr-2001	18-Jul-2001	103	VI
GS2053	plasma	26-Jun-2001	01-Aug-2001	35	V
GS2057	plasma	27-Jul-2001	14-Aug-2001	17	II
GS2075	plasma	02-Aug-2001	06-Sep-2001	34	V
GS2178	plasma	04-Feb-2002	16-Apr-2002	72	V
GS2182	plasma	26-Mar-2002	22-Apr-2002	26	IV
GS2211	plasma	06-Dec-2001	27-May-2002	171	VI
GS2275	plasma	25-Jun-2002	06-Aug-2002	41	V
GS2424	plasma	27-Nov-2002	13-Jan-2003	46	V
GS2451	plasma	28-Nov-2002	03-Feb-2003	65	V

Sample ID	Sample	Start Date#	Sample Date	DPI	Fiebig stage
GS-0005-01	Endocervical	18-Dec-2000	03-Apr-2001	105	VI
GS-0011-02	Endocervical	27-Nov-2001	20-Feb-2002	83	V
GS-0014-02	Endocervical	19-Oct-2001	26-Feb-2002	127	VI
GS-0016-03	Endocervical	07-Jan-2003	21-Jan-2003	14	I
GS-0031-02	Endocervical	18-Jan-2002	03-Apr-2002	75	V
GS-0040-03	Endocervical	13-Jan-2003	21-Feb-2003	38	V
GS-0043-02	Endocervical	22-Apr-2002	30-Apr-2002	8	I
GS-0045-01	Endocervical	14-Jun-2001	25-Sep-2001	101	VI
GS-0054-04	Endocervical	02-Feb-2004	27-Feb-2004	25	IV
GS-0067-06	Endocervical	12-Jan-2006	14-Feb-2006	32	V
GS-0090-07	Endocervical	16-Jan-2007	23-Feb-2007	37	V
GS-0181-02	Endocervical	28-Nov-2002	16-Dec-2002	18	II
GS-0220-08	Endocervical	27-May-2008	27-May-2008	0	I
GS-0308-04	Endocervical	12-May-2004	09-Nov-2004	177	I
GS-0310-05	Endocervical	07-Jun-2005	04-Nov-2005	147	VI
GS-0323-04	Endocervical	19-Aug-2004	22-Nov-2004	93	V
GS-0387-06	Endocervical	07-Nov-2006	07-Nov-2006	0	I
GS2038	Endocervical	18-May-2001	25-Jun-2001	37	V
GS2053	Endocervical	26-Jun-2001	01-Aug-2001	35	V
GS2178	Endocervical	04-Feb-2002	16-Apr-2002	72	V
GS2183	Endocervical	19-Feb-2002	23-Apr-2002	64	V
GS2424	Endocervical	27-Nov-2002	13-Jan-2003	46	V
GS3553	Endocervical	07-Mar-2005	10-May-2005	63	V

Sample IDs highlighted in bold-italic represent matched endocervical and plasma sample pairs.

Estimated time point of initial infection. The date of infection was estimated as midpoint between the date of the last negative and first positive HIV-1 test.

DPI: days post infection. DPI are calculated as number of days between Sample Date and the estimated date of infection (Start Date).
