# Supplementary material for: Higher sequence diversity in the vaginal tract than in blood at early HIV-1 infection
Source: PLoS Pathog. 2018 Jan 18;14(1):e1006754. doi: 10.1371/journal.ppat.1006754 (PMC5773221; doi:10.1371/journal.ppat.1006754)
Supplement: S1 Fig — Paired cervical and plasma consensus sequences spanning the C2-C3 region of env for the 15 individuals are presented in highlighter plots with the dominant sequence in each patient sample/compartment utilized as consensus. The nucleotide highlighter plot for the paired cervical and plasma samples are presented in panels A and B; amino acid highlighter plots in panels C and D, respectively. Nucleotide base changes (or amino acid substitutions) are highlighted in color code shown in the legend. The thickness of each line in the highlighter plot provides the amount of sequence reads as described in the legend. Panel E provides the nucleotide alignment highlighter plots for the additional HIV sequences from unpaired cervical samples from early infection whereas panels F and G display those nucleotide alignment highlighter plots for the additional unpaired plasma sample from early infection. Nucleotide (H) and amino acid (I) highlighter plots are also presented for the shorter V3 sequences from plasma samples from eight chronically infected, untreated patients from a Spanish cohort [15]. (PDF) [file ppat.1006754.s002.pdf]

A. Highlighter plot of amino acid alignment of HIV Env sequences derived from cervical tissue at early infection (paired analyses)

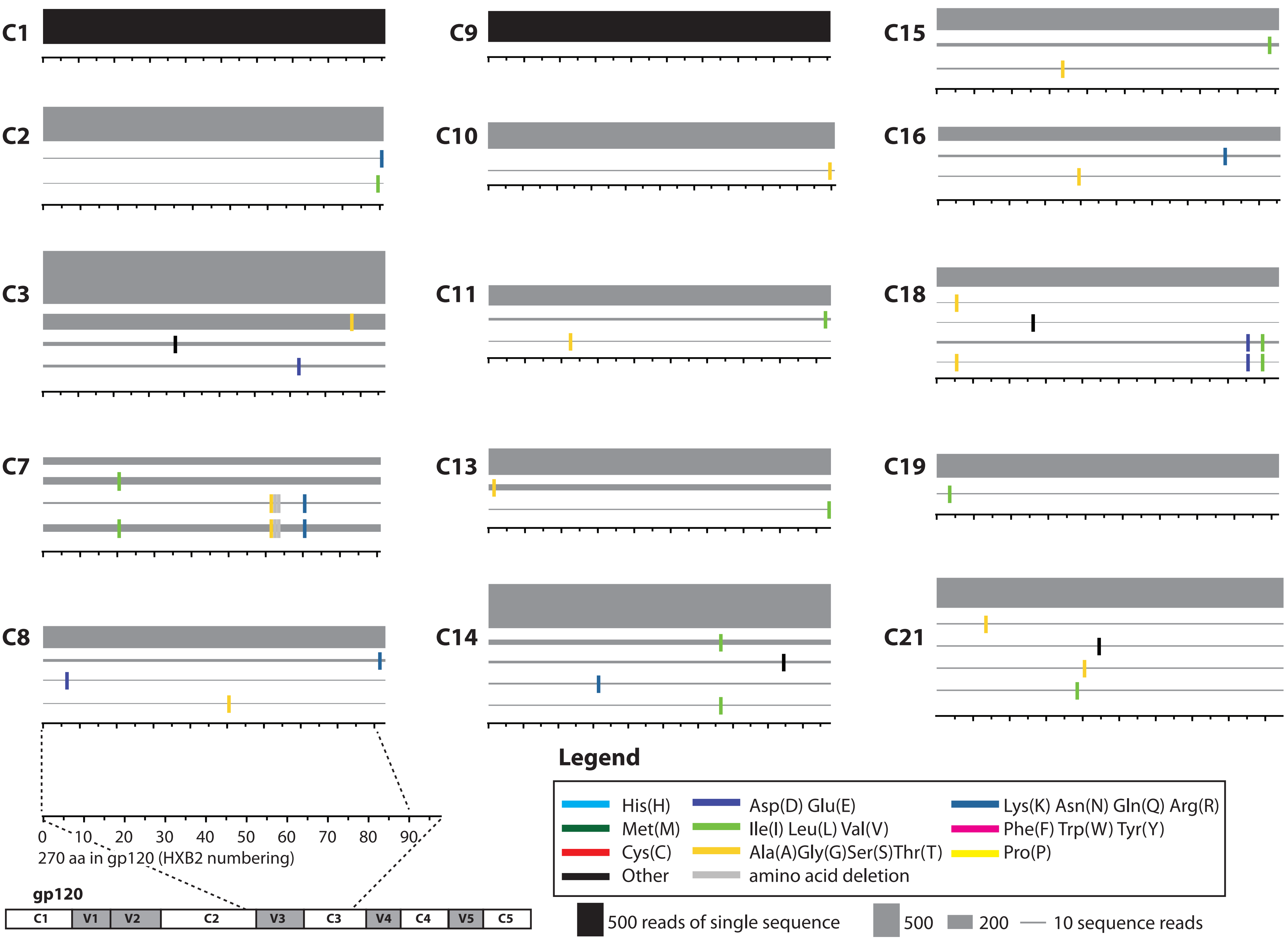

B. Highlighter plot of amino acid alignment of HIV Env sequences derived from plasma at early infection (paired analyses)

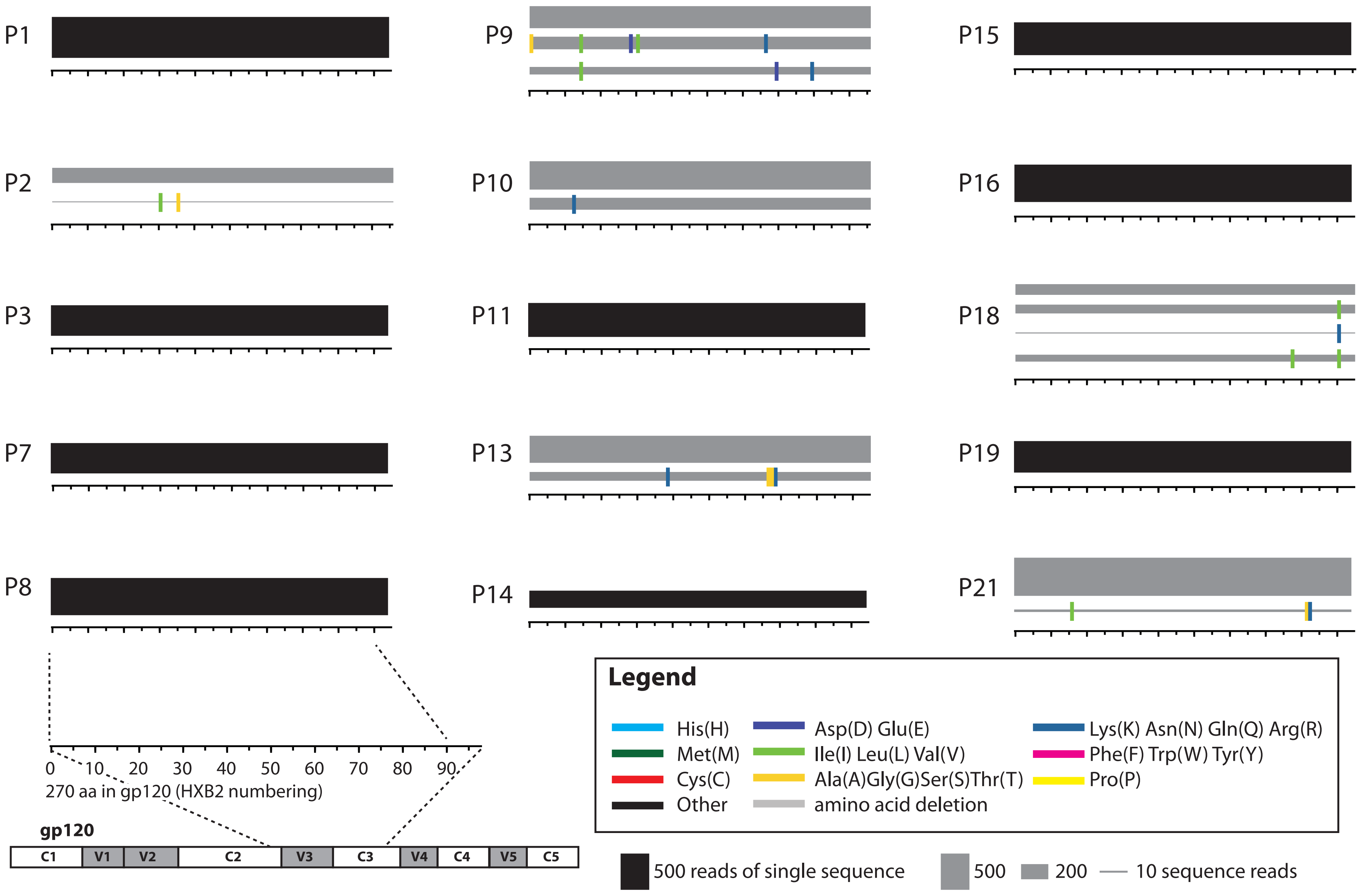

C. Highlighter plot of nucleotide alignment of HIV Env sequences derived from cervical tissue at early infection (paired analyses)

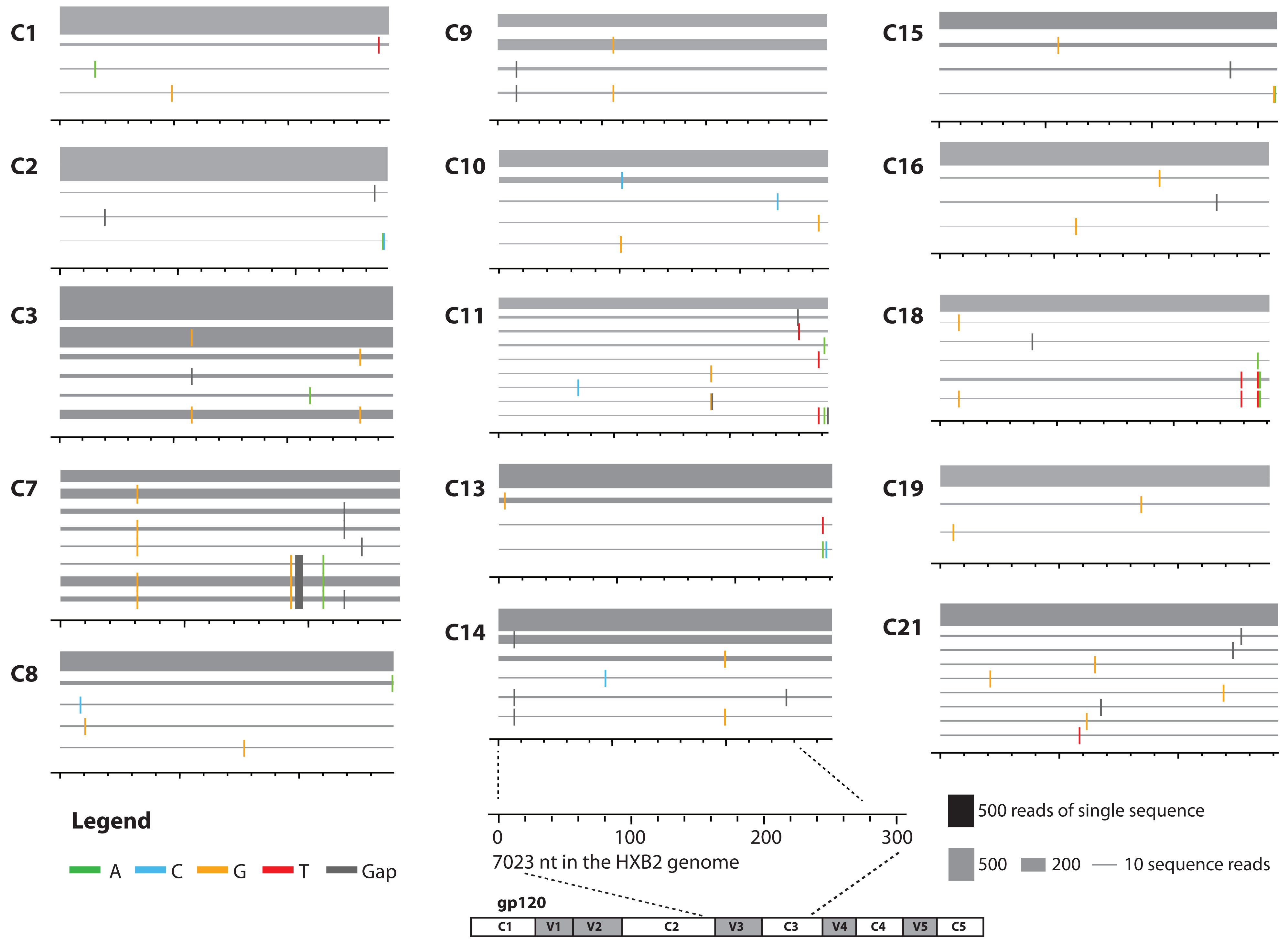

D. Highlighter plot of nucleotide alignment of HIV Env sequences derived from plasma at early infection (paired analyses)

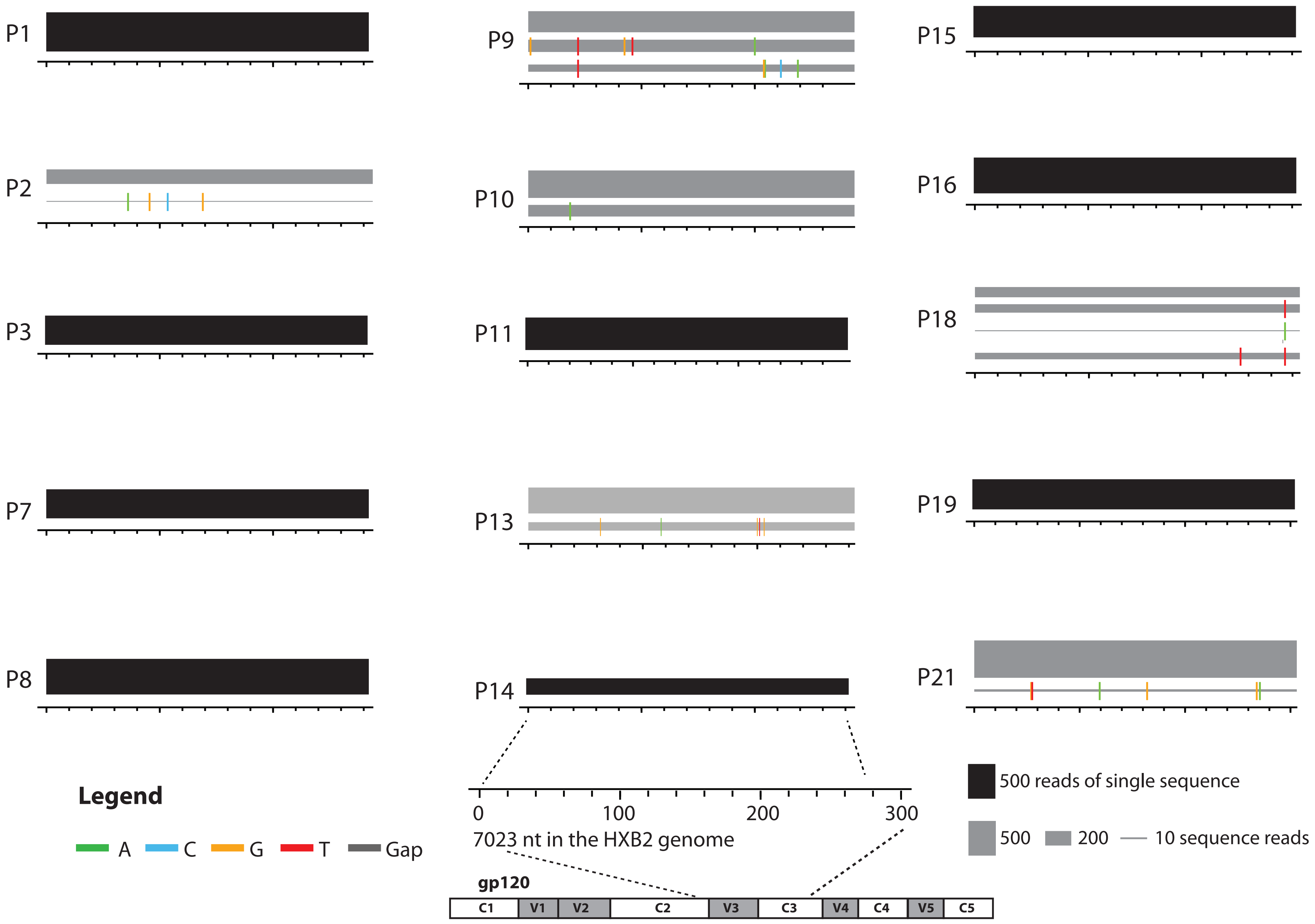

E. Highlighter plot of nucleotide alignment of HIV Env sequences derived from cervical tissue at early infection (unpaired)

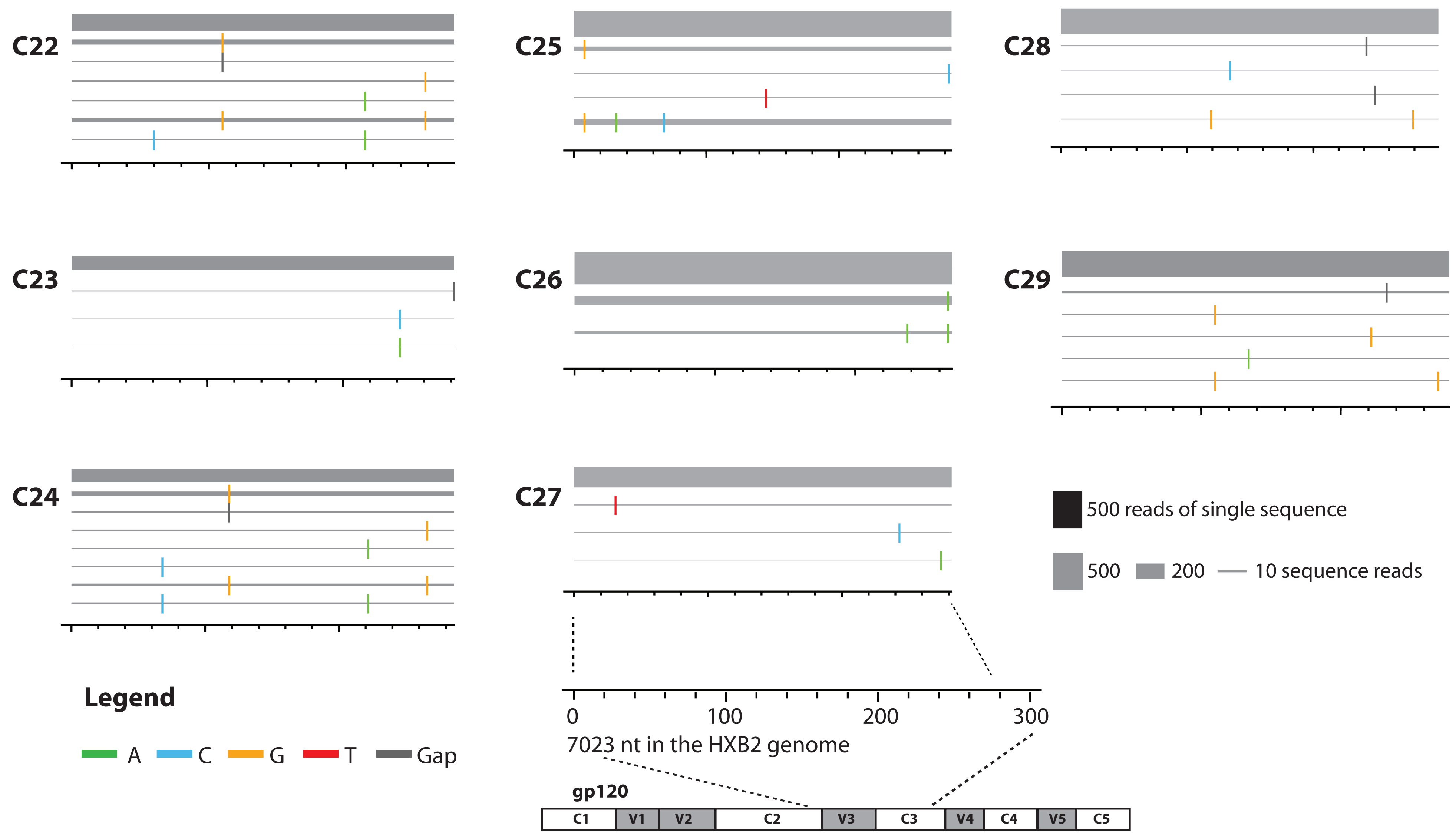

F. Highlighter plot of nucleotide alignment of HIV Env sequences derived from plasma tissue at early infection (unpaired)

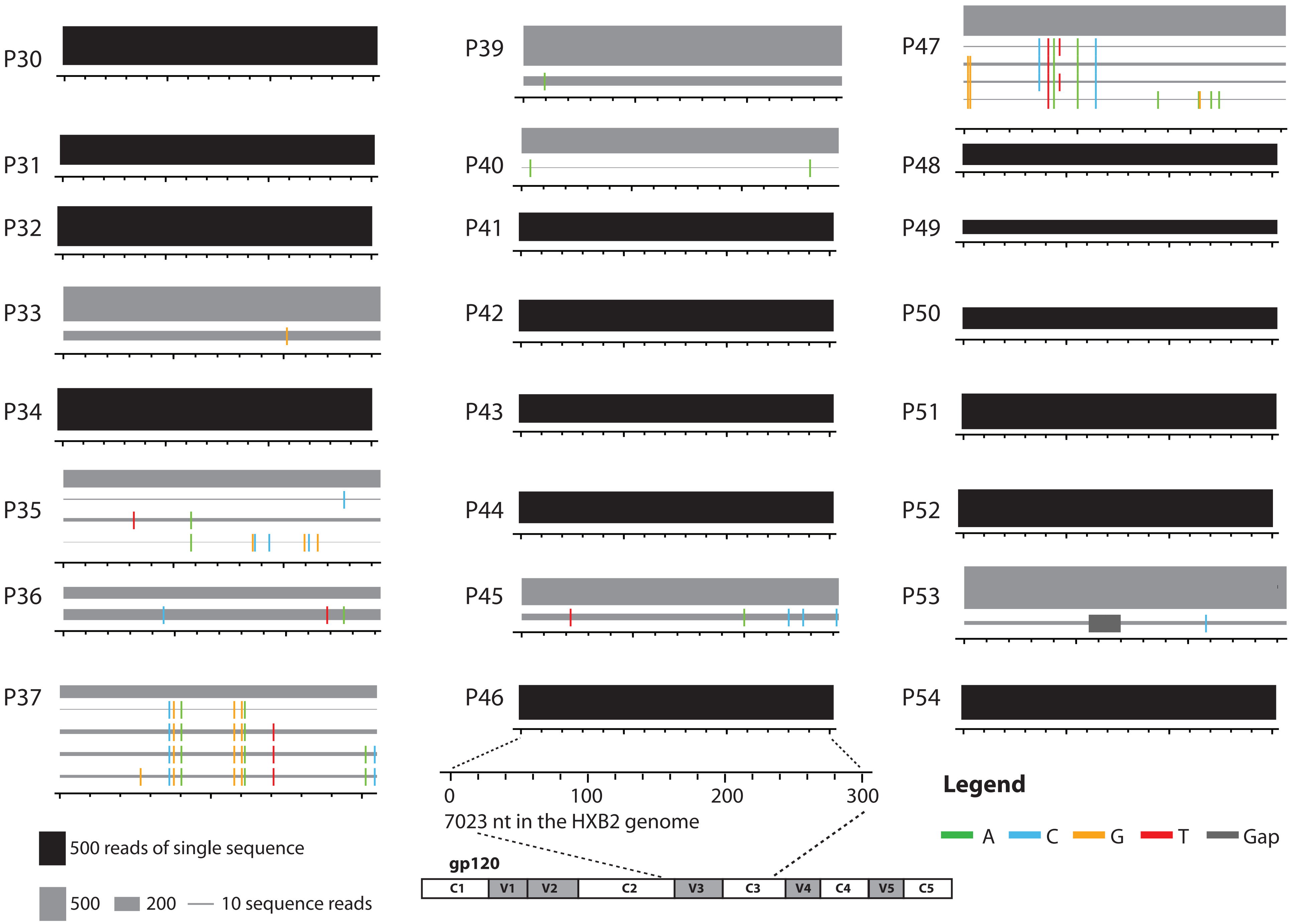

G. Highlighter plot of nucleotide alignment of HIV Env sequences derived from plasma at early infection (unpaired)

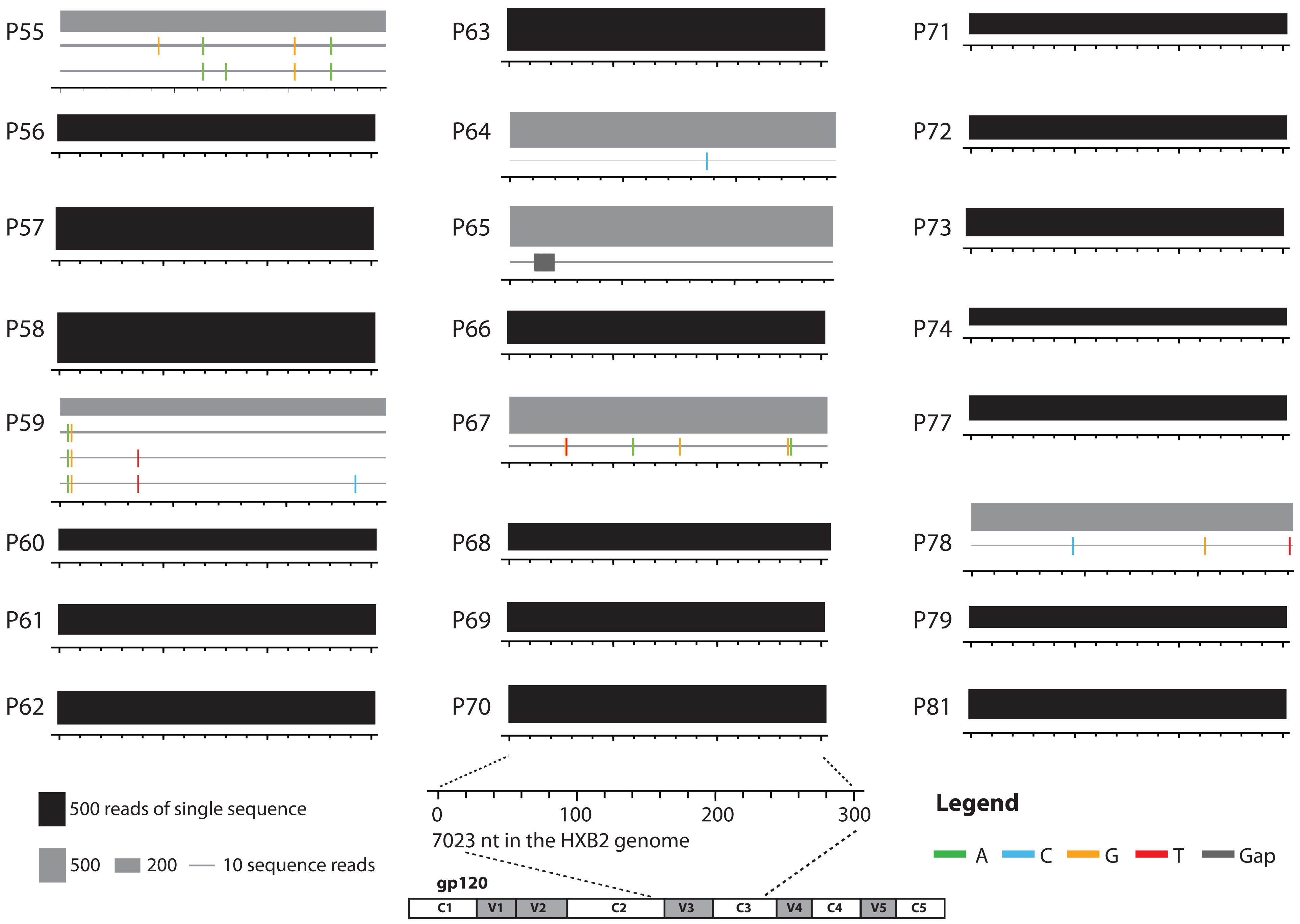

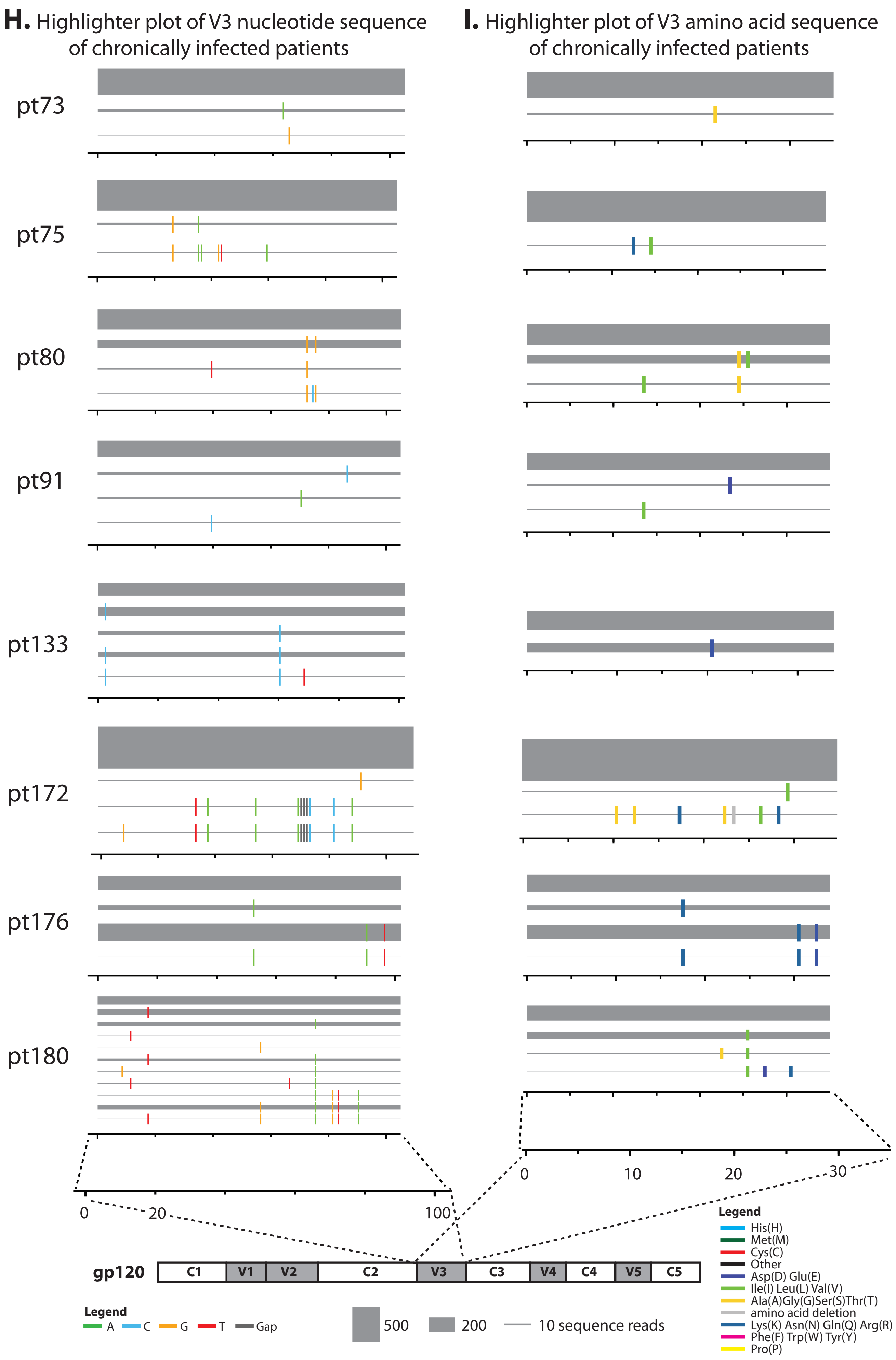

H. Highlighter plot of V3 nucleotide sequence of chronically infected patients
I. Highlighter plot of V3 amino acid sequence of chronically infected patients
pt73
pt75
pt80
pt91
pt133
pt172
pt176
pt180
0
20
100
0
10
20
30
gp120
C1
V1
V2
C2
V3
C3
V4
C4
V5
C5
Legend
A
C
G
T
Gap
500
200
10 sequence reads
Legend
His(H)
Met(M)
Cys(C)
Other
Asp(D) Glu(E)
Ile(I) Leu(L) Val(V)
Ala(A)Gly(G)Ser(S)Thr(T)
amino acid deletion
Lys(K) Asn(N) Gln(Q) Arg(R)
Phe(F) Trp(W) Tyr(Y)
Pro(P)
