# Supplementary figures and images for: Higher sequence diversity in the vaginal tract than in blood at early HIV-1 infection
Source: PLoS Pathog. 2018 Jan 18;14(1):e1006754. doi: 10.1371/journal.ppat.1006754 (PMC5773221; doi:10.1371/journal.ppat.1006754)

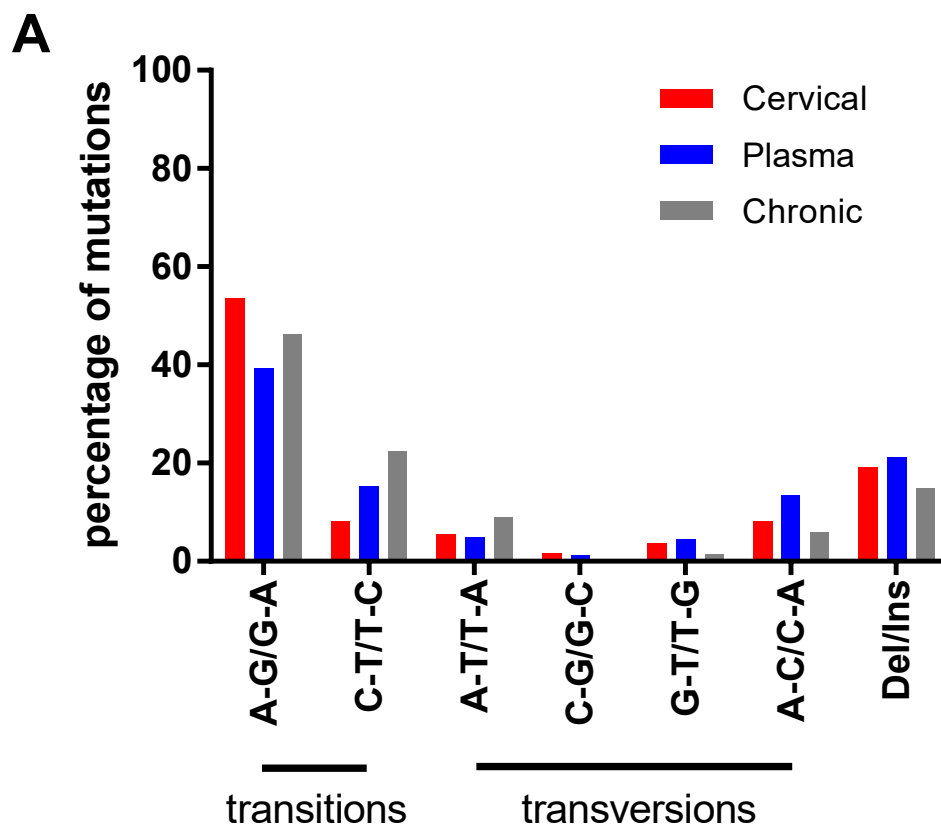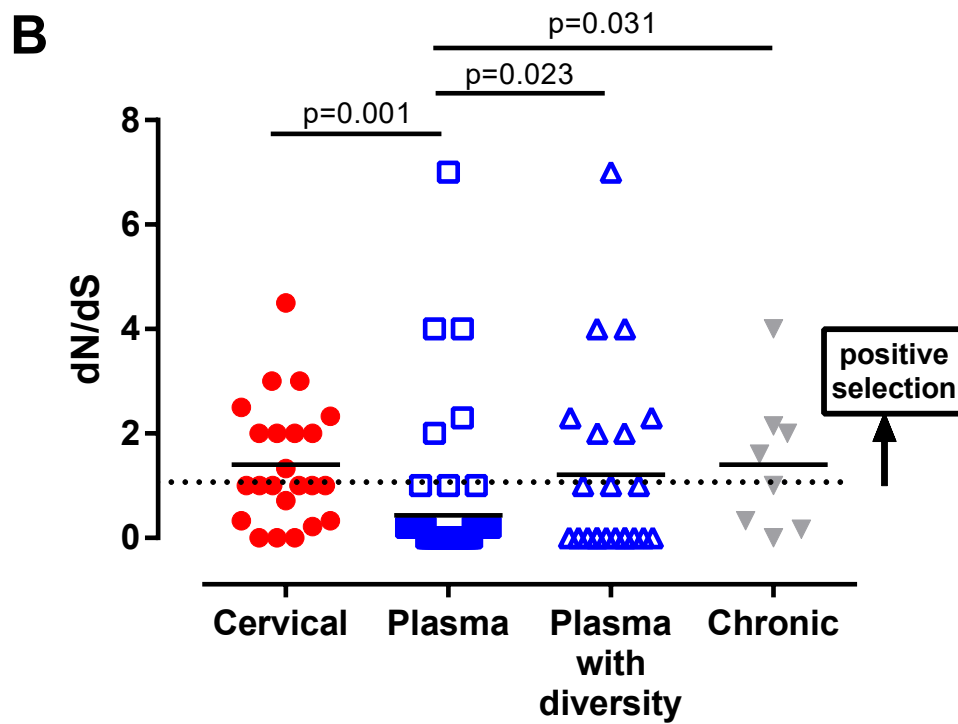

Supplement: S2 Fig — As described the percentage of A-to-G/G-to-A, C-to-T/T-to-C (transition mutations), A-to-T/T-to-A, C-to-G/G-to-C, G-to-T/T-to-G, and A-to-C/C-to-A (transversion mutations) were plotted (A) for the intrapatient HIV quasispecies in the early cervical and plasma samples as well as in the plasma samples from chronically infected patient [15]. Cervical and plasma sequences were analyzed for selective pressure by estimating the dN/dS ratio in each sample using SNAP v2.11 (B). Sequences of dS = 0 or dN = 0, resulting in dN/dS = 0 were excluded from the analysis under the label “Plasma with diversity”. Positive selection is evident in samples where dN/dS ratios are greater than 1. All substitutions were determined from the dominant HIV clone in each patient sample. Statistical significance were determined using ANOVA. (PDF) [file ppat.1006754.s003.pdf]

### A. Cervical HIV-1 V3-C3 sequences

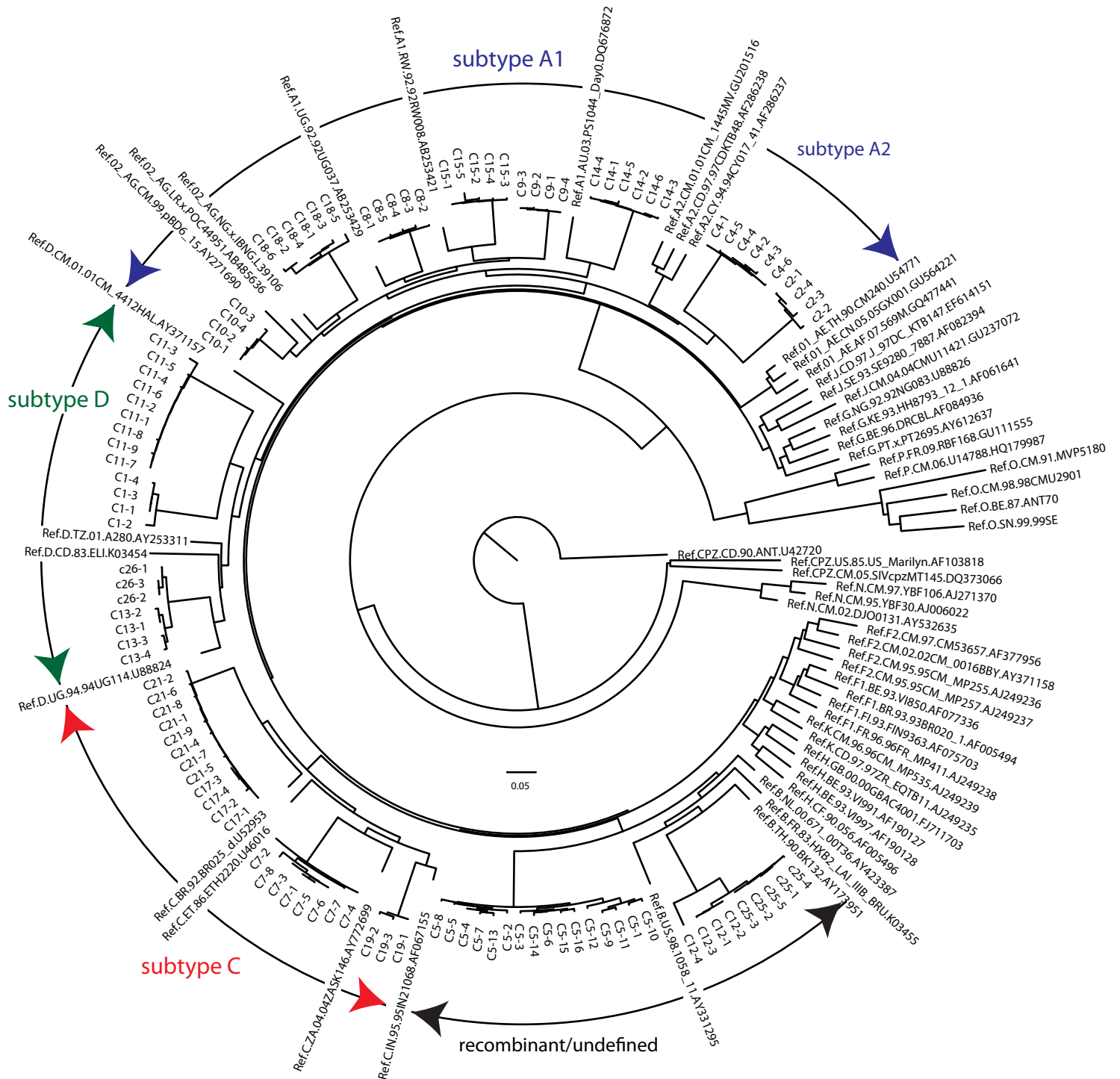

## B. Plasma HIV-1 V3-C3 sequences

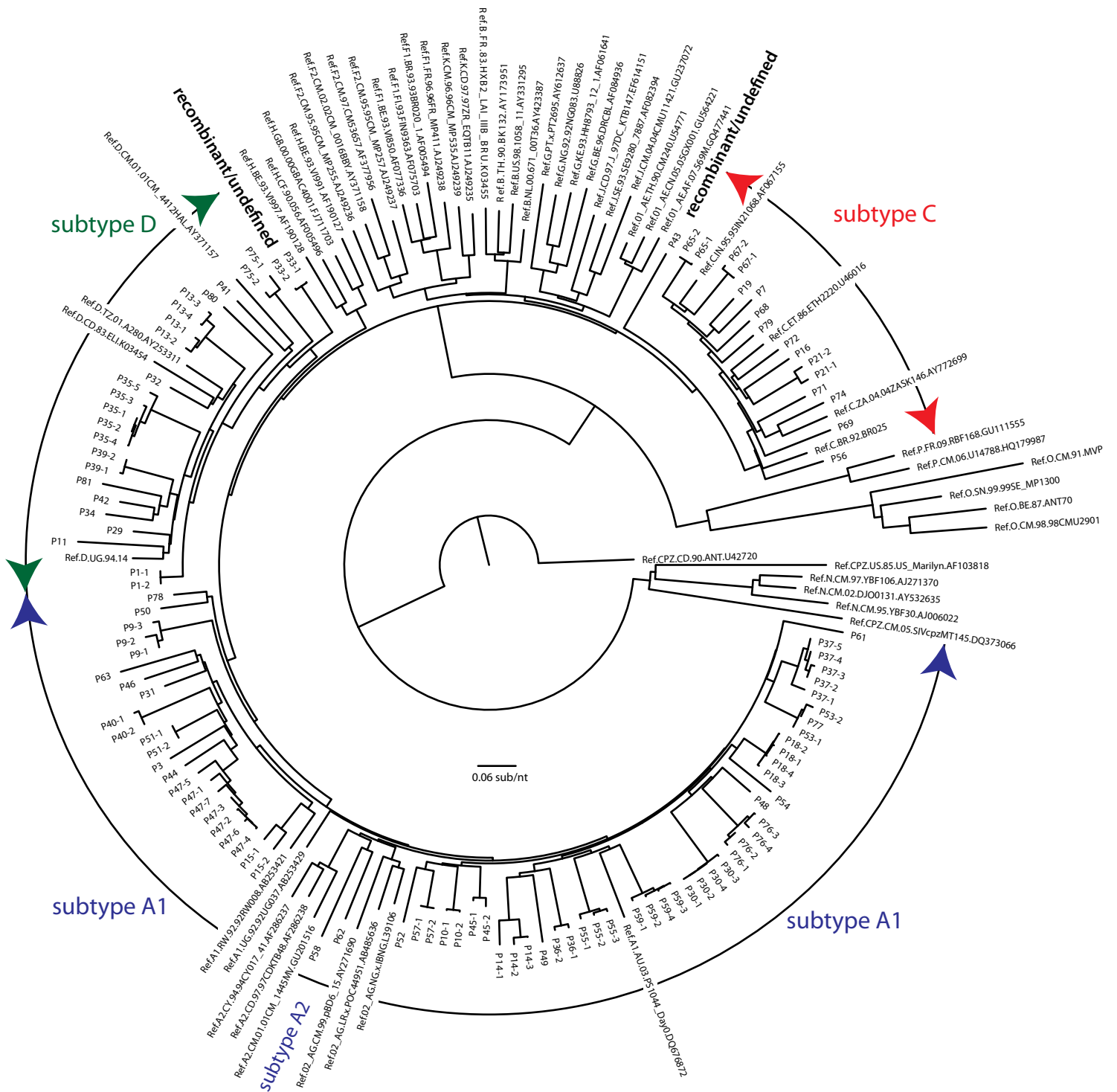

Supplement: S3 Fig — The average of 100 maximum likelihood bootstrapped trees of nucleotide sequences were generated with MEGA6, rooted to the SIVcpz CD.90.ANT sequence, and visualized with FigTree 1.4.2 to highlight sequence heterogeneity. The HIV-1 C2-V3-C3 env sequences from cervical (A) and plasma (B) samples aligned to the reference HIV sequences from the Los Alamos Sequence Database. (PDF) [file ppat.1006754.s004.pdf]

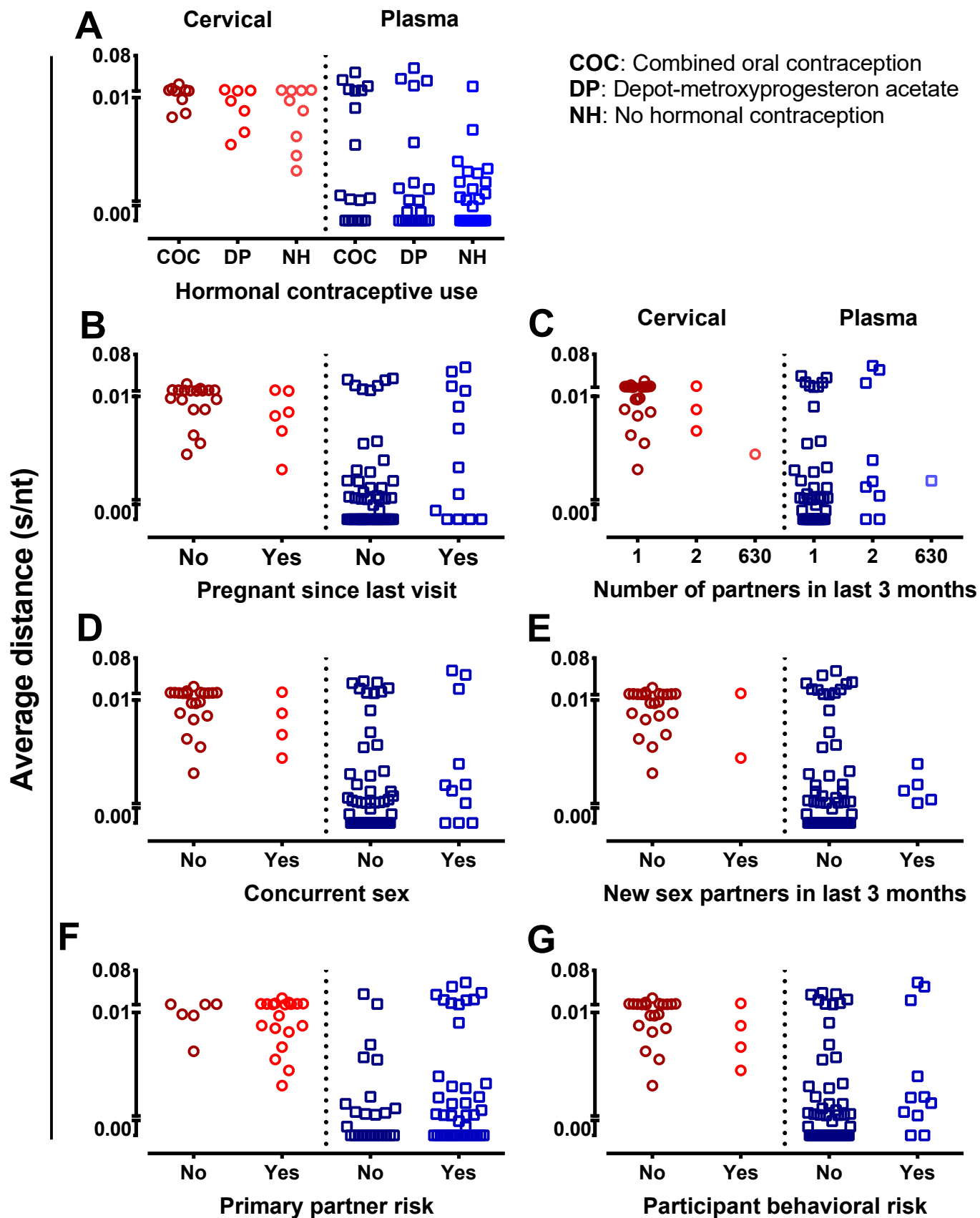

Supplement: S4 Fig — Women enrolled in the prior hormonal contraceptive study were administered combined oral contraception (COC), Depot-medroxyprogesterone acetate (DP) or did not receive any hormonal contraception (NH). Upon HIV diagnosis the women were transferred into the current study and their viral diversity in plasma and cervical mucosa evaluated based on the contraception protocol they utilized (A). The incidence of pregnancies (B), the number of sexual partners (C), concurrent sex acts (D), incidence of new sexual partners (E), the primary partner risk behavior (including the partner being HIV+, abnormal discharge from penis, weight loss, if the partner had sex with another woman or partner spends nights away from home) (F) and participant behavior risk (including having multiple partners, a new sex partner, is engaged in commercial sex work or had sex with another man in the last 3 months) (G) were also stratified based on HIV viral diversity. Statistical analysis was done using a two-tailed Mann-Whitney test. (PDF) [file ppat.1006754.s005.pdf]

Number of unique sequences

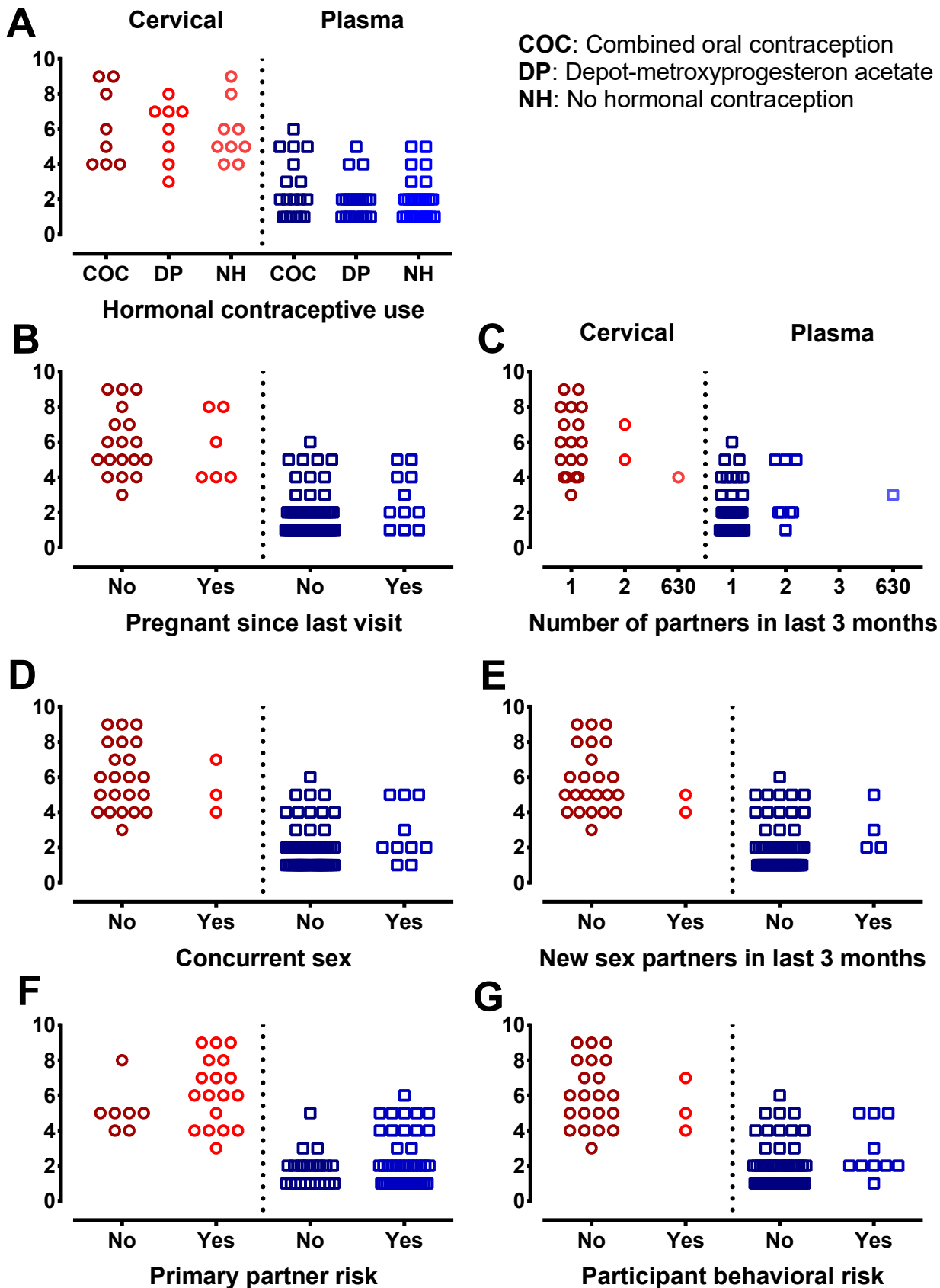

Supplement: S5 Fig — The number of unique sequences identified in cervical and plasma samples were analyzed based on clinical predictors. These included the use or absence of hormonal contraception (A), the incidence of pregnancies (B), the number of sexual partners (C), concurrent sex acts (D), incidence of new sexual partners (E), the primary partner risk behavior (including the partner being HIV+, abnormal discharge from penis, weight loss, if the partner had sex with another woman or partner spends nights away from home) (F) and participant behavior risk (including having multiple partners, new sex partner, engaged in commercial sex work or had sex with another man in the last 3 months) (G). Statistical analysis was done using a two-tailed Mann-Whitney test. (PDF) [file ppat.1006754.s006.pdf]

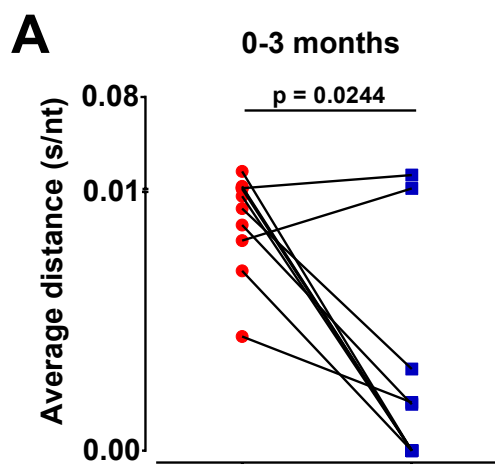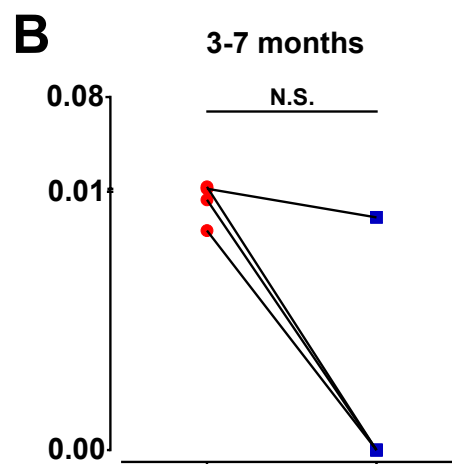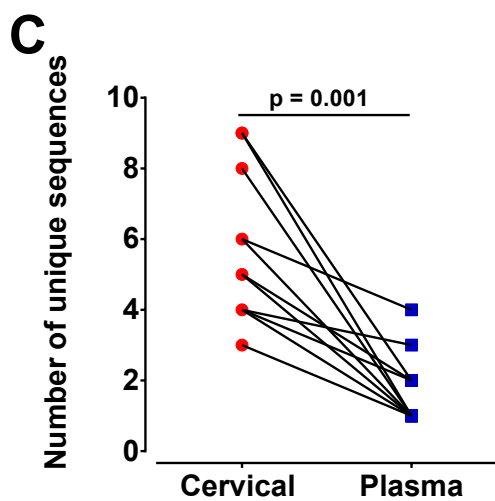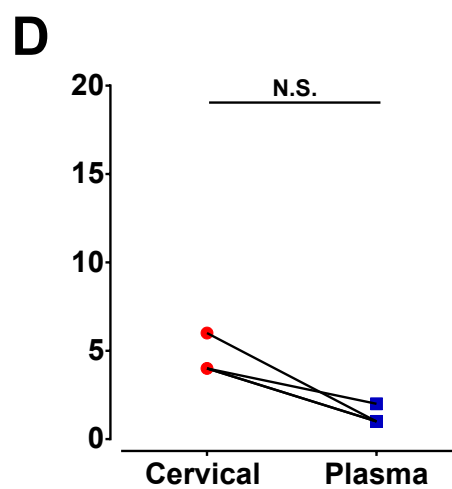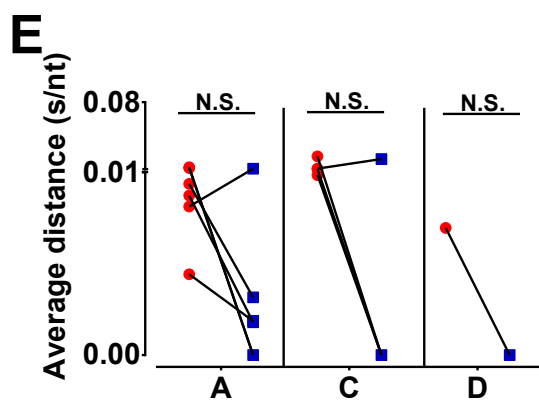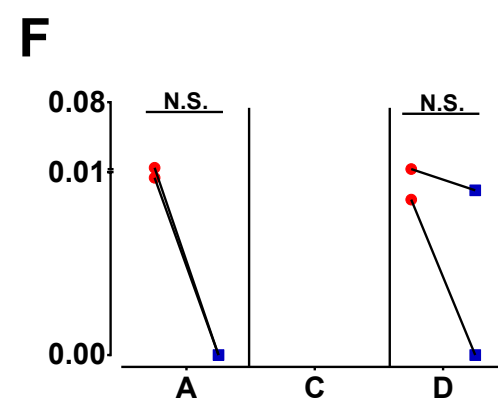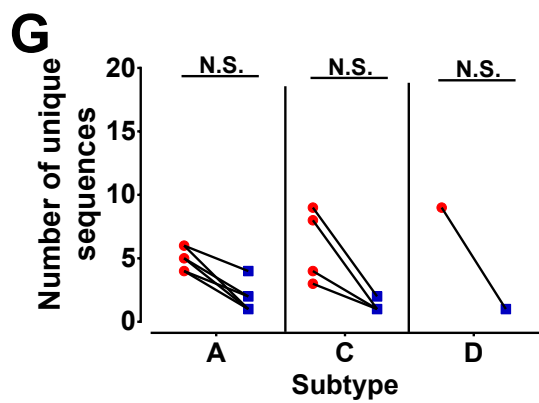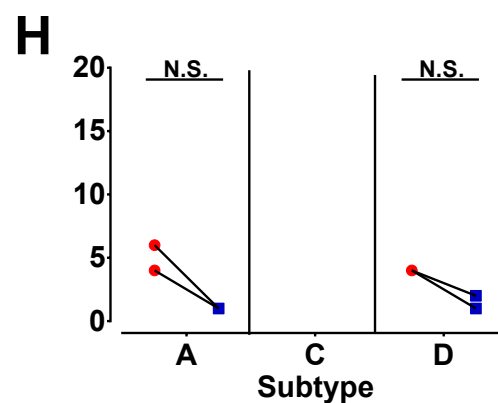

Supplement: S7 Fig — Genetic distance and number of unique sequences of paired cervical and plasma samples were grouped into very early (0–3 months, n = 14) and early infection (3–7 months, n = 7) and separated by HIV-1 subtype of infection (A, C and D) (A-H). Samples were separated by subtype A, C or D and average distance and number of unique sequences plotted as described previously (E-H). Statistical analysis for significance between cervical and plasma samples were determined using a two-tailed Wilcoxon matched-pairs signed rank test (exact p value is shown; N.S., not significant). (PDF) [file ppat.1006754.s008.pdf]

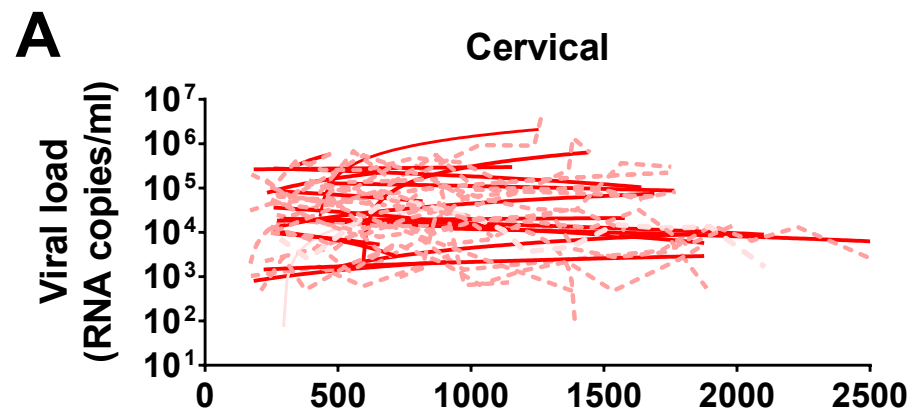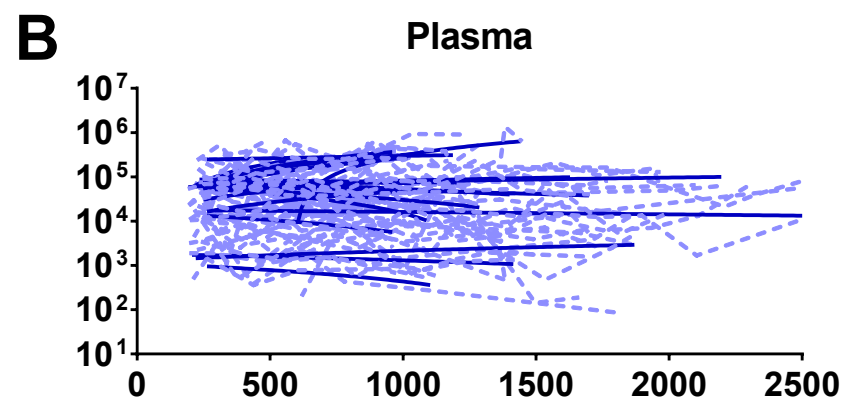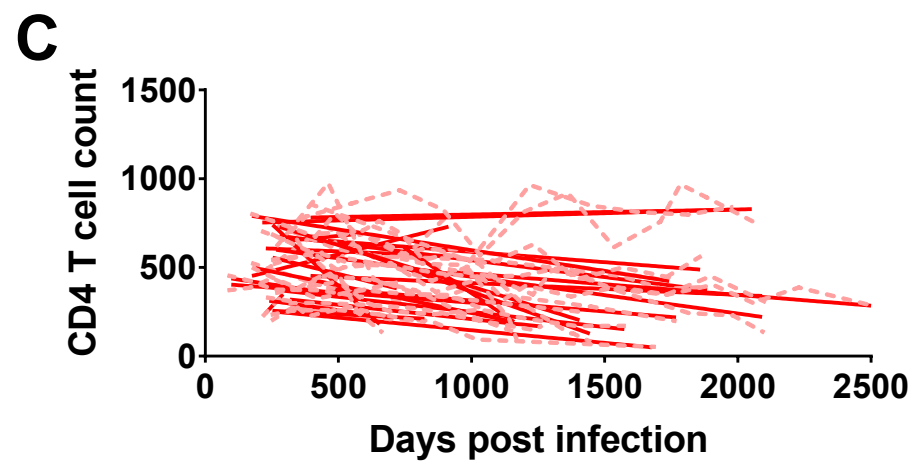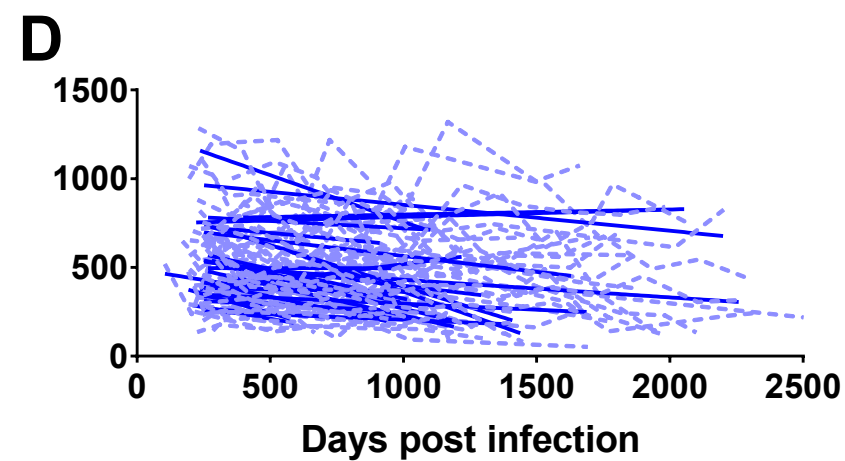

Supplement: S8 Fig — Plasma viral loads were assessed from multiple time points up to 7 years from HIV diagnosis and stratified according to linked cervical (A) or plasma samples (B). Likewise, the CD4 T cell counts were also assessed according to linked cervical (C) or plasma samples (D). Plasma viral loads were determined using a Roche Amplicor HIV-1 Monitor Test, version 1.5 while CD4 T cell counts were determined using an optimized 4-colour flow panel on a FACSCalibur flow cytometer. Linear regression analysis was performed using GraphPad PRISM 6 version 6.07. (PDF) [file ppat.1006754.s009.pdf]
