# Supplementary material for: Higher sequence diversity in the vaginal tract than in blood at early HIV-1 infection
Source: PLoS Pathog. 2018 Jan 18;14(1):e1006754. doi: 10.1371/journal.ppat.1006754 (PMC5773221; doi:10.1371/journal.ppat.1006754)
Supplement: S6 Fig — Predictors of the number of sex acts (A-D) and the number of sex acts where participants were using a condom (E-F) were evaluated based on the average genetic diversity of HIV (s/nt) in the cervical (A and E) and in the plasma (B and D) compartments or based on the number of unique HIV sequences in the cervical (C and G) and in the plasma (D and H) compartments. Each symbol represents an individual patient. Statistical significance was performed using a Spearman Rank correlation analyses. (PDF) [file ppat.1006754.s007.pdf]

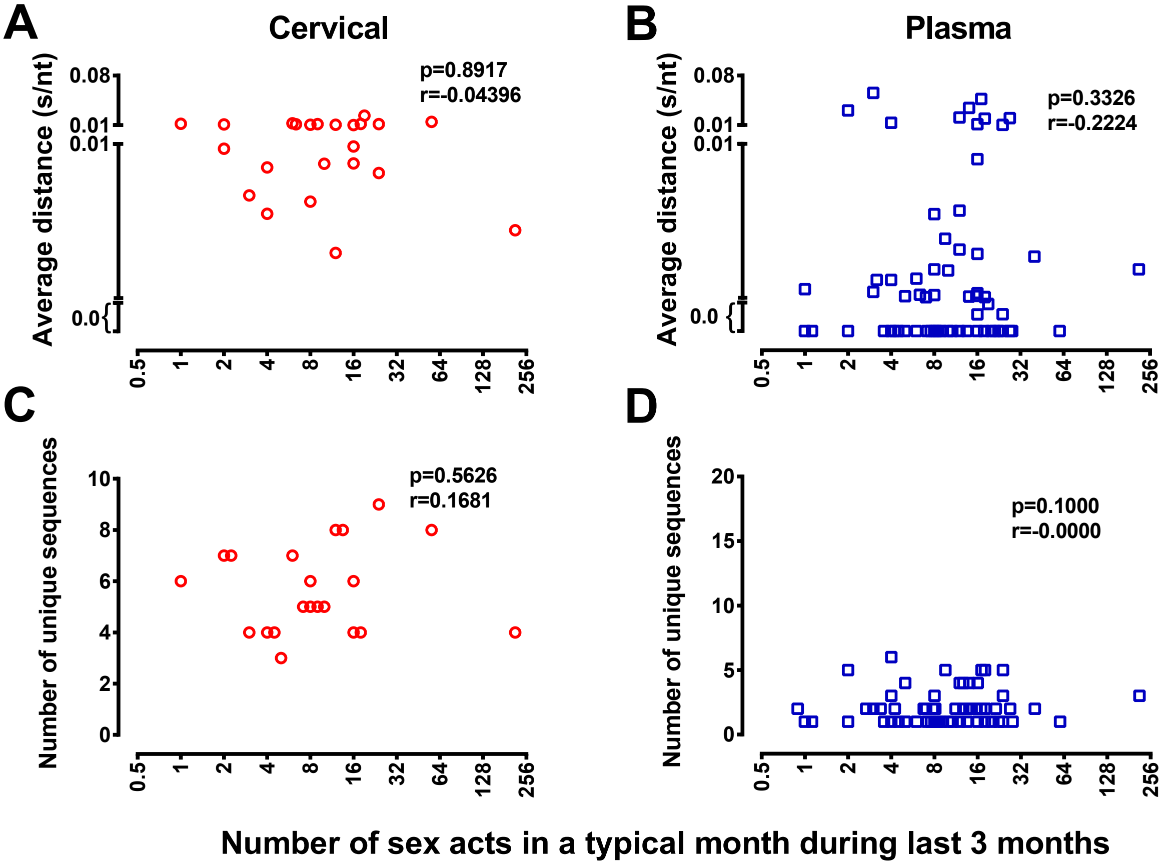
A
Cervical
B
Plasma
Average distance (s/nt)
0.08
0.01
0.01
0.0
p=0.8917
r=-0.04396
p=0.3326
r=-0.2224
C
D
Number of unique sequences
p=0.5626
r=0.1681
p=0.1000
r=-0.0000
0.5
1
2
4
8
16
32
64
128
256
Number of sex acts in a typical month during last 3 months

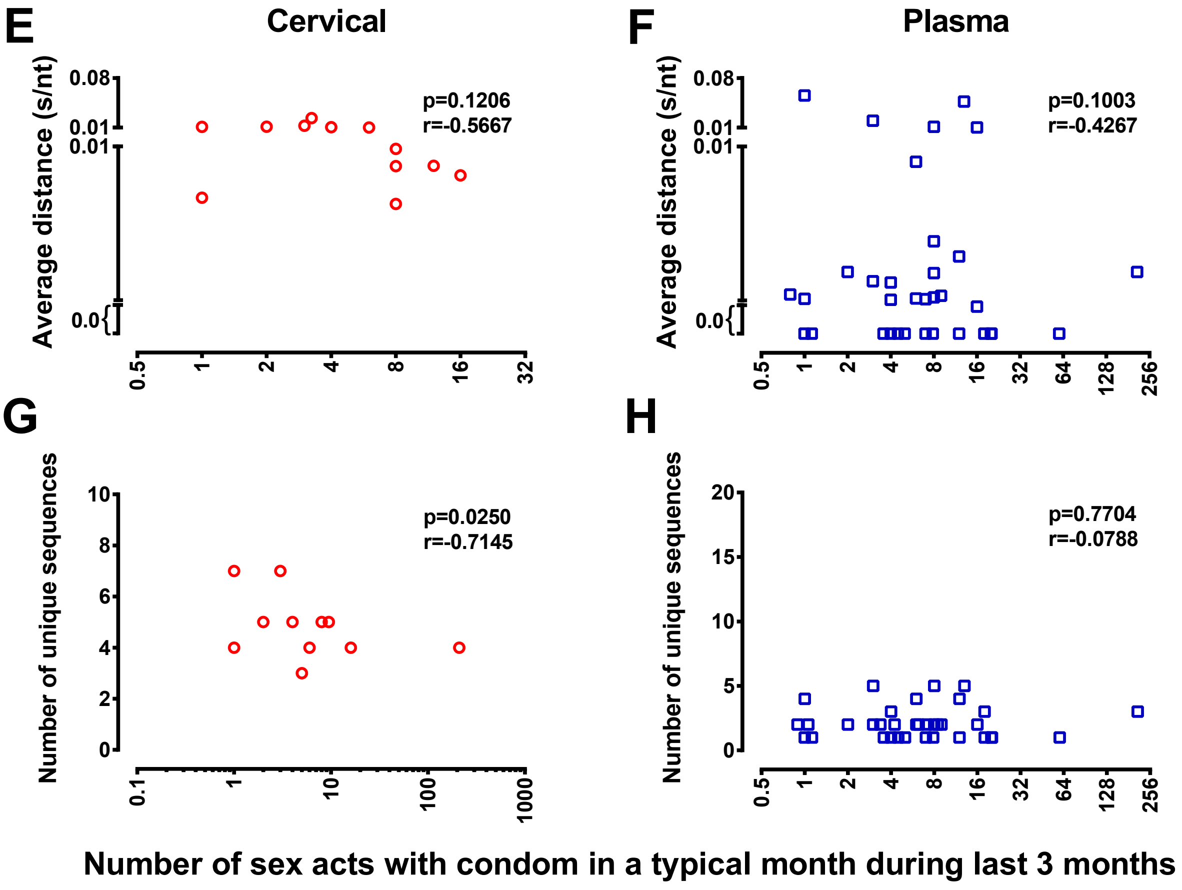
E
Cervical
F
Plasma
Average distance (s/nt)
0.08
0.01
0.01
0.0
p=0.1206
r=-0.5667
p=0.1003
r=-0.4267
G
H
Number of unique sequences
p=0.0250
r=-0.7145
p=0.7704
r=-0.0788
0.1
1
10
100
1000
Number of sex acts with condom in a typical month during last 3 months
